# An EPO‐loaded multifunctional hydrogel synergizing with adipose‐derived stem cells restores neurogenic erectile function via enhancing nerve regeneration and penile rehabilitation

**DOI:** 10.1002/btm2.10319

**Published:** 2022-05-31

**Authors:** Jun Shao, Pan Nie, Wende Yang, Rui Guo, Dongbing Ding, Rongpu Liang, Bo Wei, Hongbo Wei

**Affiliations:** ^1^ Department of Gastrointestinal Surgery The Third Affiliated Hospital of Sun Yat‐sen University Guangzhou China; ^2^ Key Laboratory of Biomaterials of Guangdong Higher Education Institutes, Guangdong Provincial Engineering and Technological Research Centre for Drug Carrier Development, Department of Biomedical Engineering Jinan University Guangzhou China

**Keywords:** adipose‐derived stem cells (ADSC), cavernous nerve injury, erectile dysfunction, erythropoietin (EPO), hydrogel

## Abstract

Neurogenic erectile dysfunction (nED) is one of the most common and intractable postoperative complications of rectal and prostate cancer surgery and sometimes accompanies patients lifelong. The transplantation of stem cells has been proved a promising way for treatment. However, the therapeutic efficacy is severely impaired by excessive cell loss and death and poor accumulation in the injury site along with the traditional implantation strategy. Herein, an EPO‐loaded multifunctional hydrogel was designed. The hydrogels' adhesive property and mechanical strength were enhanced by adding catechol‐catechol adducts, thus significantly improving adipose‐derived stem cells (ADSC) retention and rescuing cell loss in the injury site. Meanwhile, the sustained release of EPO effectively ameliorated the viability and paracrine activity of ADSC, leading to enhanced migration of Schwann cells and differentiation of PC12 cells in vivo. On a bilateral cavernous nerve injury rat model, the present stem cell‐EPO‐hydrogel implanted strategy could significantly alleviate erectile dysfunction. The higher expression of Tuj1 and lower expression of GFAP in the major pelvic ganglia (MPG) indicated the acceleration of neural differentiation while the suppressing development of astrocytes. Also, the combined therapy restored the expression levels of eNOs, nNOs, and α‐SMA in penile tissues, suggesting the rehabilitation of the penis. Further analysis of Masson trichrome staining and apoptosis evaluation of the corpus cavernosum showed the preservation of vascular endothelium content and the prevention of penile fibrosis after denervation. Overall, we believe that this combined strategy presents a promising way not only for restoring neurogenic erectile function but also for the clinical translation of stem cell therapy.

## 1 INTRODUCTION

Rectal and prostate cancers are both among the most common malignant tumors with increasing prevalence worldwide. Radical resection of the lesion is one of the most efficient treatment options. Postoperative erectile dysfunction (ED) is a significant complication and frequently occurs in male patients for the sake of inadvertent crush, tension, and resection injuries to the pelvic autonomic nerve (PAN) during the surgical procedure.^1^, ^2^ Although many advances have been made, such as more elaborate surgical anatomy and nerve‐sparing techniques, the incidence of neurogenic erectile dysfunction (nED) remains high.^3^, ^4^ Furthermore, phosphodiesterase‐5 inhibitors, which are the most popular choice for ED patients, have encountered significant challenges in the treatment of nED.^5^ Besides, the therapeutic effect of alternatives, including the intracavernous injections (ICs) of alprostadil or prosthetic implants, is too limited to cure nED. It is therefore highly imperative to explore new therapeutic strategies to improve clinical outcomes in these cases.^6^

Unlike ED resulting from aging and diabetes, nED is essentially a neurotraumatic disease. Previous studies have revealed that sufficient penile erection cannot be imagined without a sound neural network, an intact vascular system, and healthy cavernosal tissue.^7^ The major pelvic ganglion (MPG), cavernous nerve (CN), and dorsal penile nerve are the primary nerves that innervate the penis. Once an injury occurs, nerve impulse conduction is affected, and the connections between MPG/CN and penis are interrupted. In turn, denervation will induce the downstream remodeling in the penile tissues, such as the apoptosis of smooth muscle, fibrosis of the corpus cavernosum, and loss of endothelial content, resulting in inevitable nED.^8^, ^9^ As a result, optimal management of patients with nED should include timely and effective nerve regeneration and penile rehabilitation.

In the past decades, emerging evidence has proved that stem cell therapy such as adipose‐derived stem cells (ADSC) holds great potential for nerve regeneration and tissue engineering.^10^ Kim et al.^11^ reported an ADSC‐laden nanofibril composites to reconstruct cartilage and subchondral bone ECM matrices. Leong et al.^12^ prepared surface tethering of inflammation modulatory nanostimulators to ADSC for ischemic muscle repair. Yan et al.^13^ concluded that ADSC overexpressing N‐cadherin enhanced angiogenesis and cardiomyocyte proliferation after ischemic heart injury. The underlying therapeutic mechanism may be the differentiation of stem cells into specialized cell types (multipotency)^14^ or paracrine effects^15^, ^16^ that induce immune responses and stimulate revascularization and angiogenesis. Nevertheless, a clinical trial conducted by Bahk et al.^17^ demonstrated that the rigidity had increased in the penis while it was insufficient for penetration after infusion of stem cells into the corpus cavernosum. In addition, Fang et al.^18^ concluded that the red signal of PKH‐26‐labeled stem cells was only detectable in the first 2 days after periprostatic implantation (IP). It could be implied that the therapeutic efficacy was severely compromised by excessive cell loss and death and poor accumulation in the injury site accompanying traditional implantation strategy.^16^, ^19^ Hence, more advanced technologies urgently need to enhance stem cell viability for better treatment.

Hydrogels are polymeric networks that bond together by physical and/or covalent crosslinks. Due to its excellent biological properties, biodegradability, and biocompatibility, hydrogels derived from natural biomaterials have been extensively developed in tissue engineering and nerve regeneration. Meanwhile, increasing evidence has demonstrated the advantage of the combined implantation of stem cells with biocompatible hydrogels over utilizing alone, in which hydrogels provide a temporary shelter for cell adhesion, proliferation, and migration, while the implanted cells modulate the local microenvironment to compensate for the damaged tissues.^10^, ^14^, ^20^ For example, Li et al.^14^ fabricated a multifunctional hydrogel encapsulating mesenchymal stem cells (MSC) to induce neural differentiation, contributing to significant nerve regeneration in a spinal cord injury model.

Besides, due to the intricacy of the microenvironment after nerve injury such as Wallerian degeneration and inflammatory infiltration, the addition of exogenous of neuro‐protective‐related cytokines has been revealed to further improve stem cell therapeutic efficiencies, such as nerve growth factor (NGF), platelet‐derived growth factor (PDGF), brain‐derived neurotrophic factor (BDNF), NT3, and NT4.^21^, ^22^, ^23^, ^24^ As a renal hormone regulating hematopoiesis, erythropoietin (EPO) is initially applied for anemia recovery. Recently, accumulating evidence suggests its neuroprotective and neurotrophic activities mediated by EPO‐receptor (EPOR) expressed by neurons in the nervous system.^25^, ^26^ Moreover, EPO is revealed to improve the migration of Schwann cells (SCs) in the injured nerve via the local production of fibronectin.^27^ Pretreatment or modification by EPO was reported to enhance the proliferation and migration of stem cells, thus promoting its protective effects in nerve injury.^28^, ^29^ Meanwhile, EPO receptor expression was identified and localized in penile tissues and the periprostatic neurovascular bundles responsible for erectile function.^30^

**SCHEME 1:**
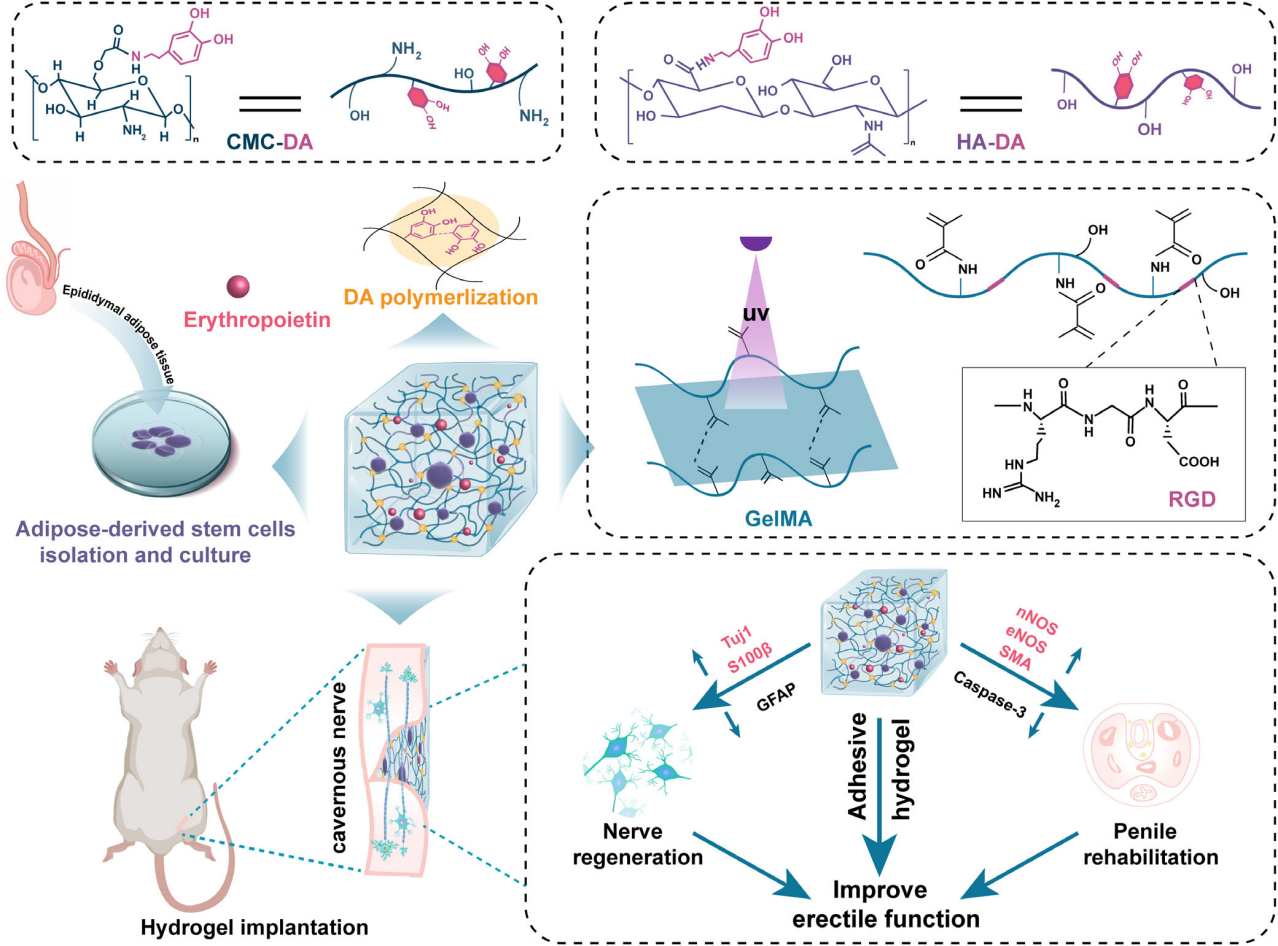
Concept illustration. GelMA is fabricated and the adhesive property is improved via DA polymerization. Then, the EPO‐loaded hydrogel synergizing with ADSC is applied to restore neurogenic erectile function induced by crush injury to bilateral cavernous nerve via enhancing nerve regeneration and penile rehabilitation

Herein, an EPO‐loaded multifunctional hydrogel was constructed for cell implantation to an nED rat model (Scheme 1). Methacrylate gelatin (GelMA) was chosen as the main matrix material for its excellent biocompatibility to simulate the biological function of the natural extracellular matrix (ECM). Meanwhile, the containing RGD motifs could interact with integrins to enhance cell adhesion.^31^, ^32^ In addition, mussel‐inspired chemistry was applied to compensate for the insufficient viscous properties of GelMA.^33^ Dopamine‐modified hyaluronic acid (HADA) and carboxymethyl chitosan (CMCDA) were further introduced to confer enhanced mechanical strength to the systems, ensuring efficient cell retention in the nerve injury site even if rats were highly active. Moreover, the sustained release of EPO improved the viability of ADSC and synergistically promoted the migration of SCs, contributing to highly efficient nerve regeneration and penile rehabilitation. On a bilateral cavernous nerve injury rat model (BCNI), the hydrogel system elicited significant restoration of erectile function. As a step toward clinical applications, the present stem cell‐EPO‐hydrogel implanted strategy provides a promising method to realize the stem cell therapies of nED.

## 2 MATERIALS AND METHODS

### 2.1 Reagents

Gelatin type A (from porcine skin), methacrylic anhydride (MA), and carboxymethyl chitosan (CMC) were purchased from Macklin Biochemical Technology Co., Ltd. (Shanghai, China). Active erythropoietin (EPO) was bought from Cloud‐Clone Crop. Hyaluronic acid (HA) was obtained from Freda Biopharma Co., Ltd. Dopamine hydrochloride (DA, purify >98%) was bought from Aladdin Biochemical Technology Co., Ltd. All reagents were of analytical grade unless otherwise noted.

### 2.2 Preparation of GelMA, HADA, and CMCDA hydrogels

GelMA was prepared as previously described with slight modifications.^32^ Briefly, 10 g gelatin power was wholly dissolved in 100 ml PBS at 50°C, then added dropwise methacrylic anhydride (MA) at a mass ratio of 0.6:1. After reaction for at least 6 h under constant agitation at 50°C, the obtained solution was processed to dialyze against deionized water for 3 days to remove all the unreacted reagents (MWCO:12–14 kDa). Finally, the GelMA solution was lyophilized at −80°C and stored at −20°C before use.

HADA was synthesized by an EDC/NHS reaction.^33^ Generally, 1 g HA was utterly dissolved in deionized water (100 ml) under a nitrogen atmosphere, followed by the supplement of 575‐mg EDC and 345 mg NHS. The mixture was stirred for 20 min before adding 569 mg DA. 0.1 M NaOH or HCl was utilized to adjust the pH value of the mixture between 5 and 6. After 12‐h reaction at room temperature, the solution was dialyzed for 3 days (MWCO: 1 kDa) to remove all impurities under acidic conditions. The final HADA solution was lyophilized at −80°C and stored at −20°C for further use.

To prepare the CMCDA conjugate, 1‐g CMC was first completely dissolved in deionized water (20 ml) at 60°C, followed by the addition of 575‐mg EDC. In addition, 345 mg NHS and 569 mg DA were weighed and dissolved in 20 ml deionized water. Then, the CMC/EDC solution was dropwise added to the DA/NHS mixture. The pH value was kept at 5, and the by‐products were removed by centrifugation after reaction in nitrogen ambient for 6 h. The supernatant was harvested and dialyzed against hydrochloric acid (pH = 5) for 24 h, then dialyzed against deionized water. The CMCDA solution was lyophilized at −80°C and stored at −20°C before use.

### 2.3 Precursor construction of the hydrogels

To construct precursors of hydrogels, the concentration of GelMA was maintained at 10% (wt/vol), with or without the supplement of 2% (wt/vol) HADA and 5% (wt/vol) CMCDA solution (3: 2: 1 by volume), followed by the addition of 0.1% (wt/vol) photo‐initiator LAP. A 365‐nm UV laser was applied for gelatinization.

### 2.4 Characterization of the hydrogels

#### 2.4.1 Physicochemical properties

Briefly, UV–vis spectrophotometer (UV‐3100PC, Mapada Instruments) and Fourier‐transform infrared (FT‐IR) spectroscopy (Bruker VERTEX70) was applied to determine the chemical structures of HA, HADA, CMC, and CMCDA. Samples were dissolved in pure water, and the wavelength was set ranging from 190 to 500 nm to obtain the absorption spectra. As for FT‐IR, the spectra were investigated over a scanning wavelength from 4000 to 399 cm^−1^ at a resolution of 4 cm^−1^. Finally, Gelatin and GelMA were dissolved in D_2_O, and the nuclear magnetic resonance (^1^H‐NMR) spectra were recorded on a 600 MHz nuclear magnetic resonance spectrometer (Bruker AVANCE).

#### 2.4.2 Swelling ability

A conventional gravimetric method was performed to assess the swelling properties. Lyophilized prepared hydrogels were weighed and recorded as *m*_*0*_. Then, the hydrogels were submerged in PBS at 37°C, and the swollen weight was measured at selected time intervals and recorded as *m*_*1*_. The swelling rate was calculated according to the following equation:

$$\text{Swelling rate}\;\left(\%\right)=\frac{m_{1}-m_{0}}{m_{0}}\times 100\%.$$

#### 2.4.3 Mechanical properties

Rheological tests were performed using a rotary rheometer (Kinexus) at 25°C with plate geometry (plate diameter: 25 mm). Frequency sweep tests were conducted to analyze the stability of the hydrogels over a range frequency from 0.1 to 10 Hz and a 5% constant strain. Time sweep tests were carried on to observe the stiffness of the hydrogels at a 1 Hz frequency and a 1% constant strain. Besides, a Bose ElectroForce 3200 instrument (Bose) was utilized to analyze the compressive stress–strain and adhesive property. For the compression test, the 6‐mm‐high hydrogels were subjected to compression until crushing. For the adhesion test, 200‐μl hydrogels were applied between two pieces of fresh porcine skin (adhesion area: 1 cm × 1 cm).^33^

#### 2.4.4 In vitro degradation analysis

Enzymatic degradation experiments were performed to systematically evaluate the degradation behavior of hydrogels. Briefly, the equal volume hydrogels were immersed in 20 ml PBS, with or without the addition of 1000 U/ml lysozyme. The solution system was kept at 37°C under constant agitation at 100 rpm. At the selected time intervals, samples were taken out and lyophilized. The pore structure and morphology of the lyophilized hydrogels were determined by an S‐3400 scanning electron microscope (Hitachi). The weights were obtained, and the degradation rate was calculated by the following equation:

$$\text{Degradation rate}\;\left(\%\right)=\frac{w_{0}-w_{1}}{w_{0}}\times 100\%,$$

where *w*_0_ and *w*_1_ represent the dry weight of the initial hydrogels and the remaining hydrogels at different time point, respectively.

### 2.5 EPO integration and in vitro release behavior of EPO

Fifty‐microliter sterile PBS buffer containing EPO (10,000 IU/ml) was mixed with 950 μl of GelMA/HADA/CMCDA pre‐gel solution completely at 37°C. EPO 500 IU/ml concentration was reported to display the most potent protective effect to stem cells.^28^ Five‐hundred microliter EPO‐loaded hydrogels (GelMA/HADA/CMCDA/EPO) were then transferred to a centrifuge tube to analyze the sustained release of EPO. After gelation, 5 ml PBS buffer was used to immerse the EPO‐loaded hydrogel shaken at 100 rpm at 37°C. PBS supernatant of 0.5 ml was collected and replaced with an equal volume of fresh PBS buffer at indicated time intervals. The concentration of EPO was detected by enzyme‐linked immunosorbent assay kits (Meimian Biotechnology Co. Ltd.).

### 2.6 Cell isolation, culture, and identification

As published previously, primary adipose‐derived stem cells (ADSC) were isolated from epididymal adipose tissue of 2‐week‐old male Sprague–Dawley rats.^34^ Then, ADSC was resuspended in low‐glucose Dulbecco's modified Eagle's medium (DMEM) supplemented with 10% fetal bovine serum (FBS), 1% penicillin, and 1% streptomycin and incubated at 37°C in a humidified atmosphere with 5% CO_2_. The culture medium was changed every 3 days, and all the cells used in experiments were at Passages 3–5. Flow cytometry was performed to identify the obtained cells. The following PE‐labeled antibodies were used: CD11b, CD45, CD14, CD29, CD44, and CD90. The data were determined by a flow cytometer (BD Bioscience). Additionally, adipocytes and osteoblasts differentiation of ADSC was further observed by culturing in a special medium. Alizarin red S was used for osteoblasts staining, and oil red O was applied for adipocytes staining.

SCs were isolated from the sciatic nerve of 2‐week‐old SD rats. First, the rats were killed, and the bilateral sciatic nerves were collected. Then, the nerve bundle was cut into ≈3‐mm segments and incubated in high‐glucose DMEM supplemented with 10% FBS at 37°C in a humidified atmosphere containing 5% CO2. Five micrograms per milliliter of cytarabine was used to purify SC. Immunofluorescence staining for S100β was performed for the identification of SC.

PC12 cells (rat adrenal pheochromocytoma‐derived cell line) were obtained from Zhongqiao Xinzhou Biotechnology Co., Ltd., and cultured in DMEM supplemented with 10% FBS for proliferation.

### 2.7 Biocompatibility test in vitro

Calcein‐AM/PI double staining, cytoskeleton staining, and cell counting kit‐8 test were utilized to assess the cytotoxicity of hydrogels on ADSC. Before seeding cells, the 35‐mm confocal dishes (Nest, 801002) were precoated with 100 μl of GelMA, GelMA/HADA/CMCDA, and GelMA/HADA/CMCDA/EPO pre‐gel solution, respectively. After being cured for 20 s by a 365‐nm UV laser, 200‐μl cell suspension of ADSC was seeded on the hydrogels (1 × 10^4^ cells/dish). One‐milliliter culture medium was then supplemented and co‐cultured for different time intervals (1, 3, and 5 days). At each time indicated, the cells were washed with PBS three times, and the live/dead reagents (Calcein‐AM and PI) were added. After incubation for 30 min, the survival and growth of ADSC were visualized under a confocal laser scanning microscope (CLSM, Olympus FV 3000). Meanwhile, to observe the cell morphology, the cell attached‐hydrogels were fixed with paraformaldehyde (4%), followed by added with TRITC phalloidin for staining F‐actin and DAPI for staining the nuclei. Additionally, a CCK‐8 assay was performed to assess the cell viability according to the protocol. The absorbance value was measured at λ = 450 nm.

### 2.8 ADSC encapsulation, viability, and spreading

ADSC encapsulation into the hydrogels was performed on previously published protocols with slight modifications.^35^ Firstly, 50‐μl cell suspension of ADSC (1 × 10^7^ cells/ml) in the logarithmic growth phase were added to 950 μl of GelMA, GelMA/HADA/CMCDA, and GelMA/HADA/CMCDA/EPO pre‐gel solution, respectively. After mixing well by pipetting, 300 μl of the above pre‐gel solutions were coated on the 48‐well plate, and then a hydrogel was polymerized by UV irradiation. The cell‐encapsulated hydrogels were co‐cultured in low‐glucose DMEM. After incubated for different time intervals (1, 3, and 5 days), the hydrogels were transferred to 35‐mm confocal dishes and gently rinsed with PBS three times. Similarly, the live/dead staining assay and cytoskeleton staining assay were performed the same as described in the *Biocompatibility test* in vitro section to observe the spatial distribution, viability, and morphology of the cells loaded in the hydrogels. CCK‐8 assay was utilized to determine the viability of the encapsulated cells.

### 2.9 Chemotaxis of SC

The transwell experiment was further employed to determine the effect of different cell co‐culture systems on the chemotaxis of SC. The cells of the 24‐well plate were randomly divided into ADSC, pre‐coated GelMA/HADA/CMCDA + ADSC, pre‐coated GelMA/HADA/CMCDA/EPO, and pre‐coated GelMA/HADA/CMCDA/EPO + ADSC (1 × 10^4^ cells/well). Fresh medium without any supplement was set as a control group. After co‐culture for 3 days, 200‐μl cell suspension of SC (1 × 10^4^ cells) was seeded in the upper chamber (8‐μm pore size, Corning). After incubation for 48 h, cells remaining on the upper membrane were removed with cotton swabs, and the migrated cells under the membrane were fixed with 4% paraformaldehyde and stained with crystal violet solution for 20 min. Finally, the upper chamber was gently washed in PBS, and the immigrated cells were observed under an inverted microscope. The cell numbers of three randomly chosen fields were counted.

### 2.10 Enzyme‐linked immunosorbent assay

The enzyme‐linked immunosorbent assay (ELISA) was performed to investigate the paracrine response of ADSC to hydrogels. Briefly, 100‐μl pre‐gels with or without EPO were added to each well of the 24‐well plate and gelated before the experiment, and the well without any supplement was set as control. Then, ADSC was seeded into the 24‐well plate at the density of 1 × 10^4^ cells/well and cultured in a fresh medium at 37°C. After co‐culture for 3 days, the supernatant of each well was harvested, and the level of neuro‐protective related cytokines (VEGF, BDNF, NGF, and PDGFα) was determined by ELISA kits (Meimian Biotechnology Co. Ltd.). Three independent experiments were conducted for statistical analysis.

### 2.11 Neurite growth on PC12 cells

Good differentiated PC12 cells demonstrate similar morphology, physiology, and biochemistry to nerve cells. Therefore, the supernatants of different cell co‐culture systems mentioned in the 2.9 section were harvested to culture PC12 cells. After different time intervals, PC12 cells were stained with Calcein‐AM and observed under a fluorescence microscope. The neurite length and growth ratio of PC12 cells were counted to assess the neurite growth. Specifically, 100 cells were randomly selected, and the length of neurites was measured by FIJI/ImageJ software. Cells were divided into four types according to the ratio of synapses with cell size: no neurite (L0), neurites length shorter than the size of the cell body (L1), neurites length between the original and twice of the cell body (L2), neurites length between twice and three times of the cell body (L3), and neurites length over three times of the cell body (L4).^36^

### 2.12 PKH26 for cell labeling and tracing

To investigate the fate of the implanted ADSC in vivo, PKH26 dye (Sigma Chemical Co.) was used to stain the cells according to the protocol. Then, the labeled cells were encapsulated into GelMA/HADA/CMCDA/EPO hydrogel for implantation of BCNI rats. The PKH26 labeled cells in the hydrogels and MPG tissues were determined under a fluorescence microscope at different time intervals.

### 2.13 Surgical procedure and treatments

All animal experiments were approved by the Ethics Committee of the Institutional Animal Care and Use Subcommittee of the Third Hospital of Sun Yat‐sen University. To establish the bilateral cavernous nerve injury (BCNI) model, 8‐week‐old Sprague–Dawley male rats were prepared and anesthetized with 2.5%–3% isoflurane. Next, the MPG/CN were exposed posterolaterally on both sides of the prostate.^37^ The bilateral CN were crushed 5 mm away from MPG as previously described.^34^, ^37^ After that, rats were randomly divided into five groups with different treatments (*n* = 5): (1) PBS group: 50 μl PBS was injected into the crush area on each side; (2) Gel group: 50 μl GelMA/HADA/CMCDA was applied for treatment on each side; (3) Gel/EPO group: 50 μl GelMA/HADA/CMCDA/EPO was used on each side; (4) Gel/ADSC group: 50 μl GelMA/HADA/CMCDA containing 1× 10^6^ ADSC; (5) Gel/EPO/ADSC group: 50 μl GelMA/HADA/CMCDA/EPO containing 1× 10^6^ ADSC was implanted on each crush side. Finally, experimental animals were kept for further evaluation. For the sake of description, Gel represents GelMA/HADA/CMCDA hydrogel.

### 2.14 Erectile function evaluation

Four weeks after surgery, both intracavernous pressure (ICP) and mean arterial blood pressure (MAP) were recorded continuously to evaluate the erectile function of animals. After anaesthetization, the right carotid artery was exposed through a midline incision from the neck to the upper thorax, and a heparinized (250 U/ml) silastic cannula connected with a three‐way stopcock was inserted to record the MAP. Similarly, a heparinized butterfly needle (24‐G) connected to a pressure transducer was implanted in the corpus cavernosum to measure the ICP. To elicit erection, a bipolar electrode with a 5 V, 20 Hz electric stimulus was applied to stimulate the CN directly. All data were recorded by a BL‐420s Biological Functional System (Chengdu Taimeng Technology, Ltd.). After the experiment period, penile tissues and MPG/CN were harvested for further analysis.

### 2.15 Histological staining analysis

Freshly dissected penile and MPG tissues were harvested and fixed with 4% paraformaldehyde for further histological staining analysis. Masson trichrome staining was first carried out to determine smooth muscle and collagen expression levels in penile tissues. A 5‐μm slice was prepared and stained following the manufacturer's instructions, and the smooth muscle was stained red while the connective tissue appeared blue. For immunofluorescence staining, the penile sections were incubated with primary antibodies including α‐SMA (1:300, Servicebio, GB111364), eNOS (1:500, Abcam, ab66127), nNOS (1:500, Servicebio, GB11145), Caspase‐3 (1:200, Servicebio, GB11009‐1), and the MPG sections were covered by primary antibodies including Tuj1 (1:2500, Servicebio, GB11139), and GFAP (1:2500, Servicebio, GB11096) at 4°C overnight. After rinsing the slices with PBS, secondary antibodies were adopted for 1‐h immersion. Nuclei were stained with DAPI. Images were observed, and the interesting areas were captured under a confocal laser scanning microscope (Zeiss LSM 710) and a fluorescence microscope (NIKON, Ti2). FIJI/ImageJ software (National Institutes of Health) was used to carry out image analysis.

### 2.16 Western blot analysis

Relative expression levels of the expected proteins in penile tissues were detected by western blot analysis as previously described.^34^ In brief, a portion of penile tissues was lysed in RIPA buffer containing phenylmethylsulfonyl fluoride. The protein was then harvested, and the concentrations were quantified by the BCA assay. Next, equal amounts of protein were loaded in 10% sodium dodecyl sulfate–polyacrylamide gel electrophoresis and electrically transferred onto a polyvinylidene fluoride membrane. Afterward, the membrane was blocked with 5% skim milk and incubated with primary antibodies including α‐SMA (1:1000, BIOSS, bs‐10196R), eNOS (1:1000, Abcam, ab66127), nNOS (1:1000, Servicebio, GB11145), Caspase‐3 (1:1000, Affinity Biosciences, AF6311), BAX (1:1000, Servicebio, GB11690), Bcl‐2(1:1000, Proteintech, 26,593‐1‐AP), and GAPDH (1:1000, Proteintech, 60004‐1‐lg) at 4°C overnight. Finally, secondary antibodies were used for hybridization, and the obtained membrane was imaged under a molecular imager ChemiDoc XRS + system (BIO‐RAD). FIJI/ImageJ software was applied to determine the integrated density.

### 2.17 Statistical analysis

All data were analyzed using SPSS 26.0 software and displayed as mean ± SD. Unpaired Student's *t*‐test was performed for comparisons between two groups, whereas one‐way analysis of variance (ANOVA) followed by post hoc Bonferroni analysis was applied for comparing multiple groups. Statistical significance is demonstrated as (*) *p* < 0.05, (**) *p* < 0.01, and (***) *p* < 0.001.

## 3 RESULTS AND DISCUSSION

### 3.1 Chemical construction of hydrogels

**FIGURE 1:**
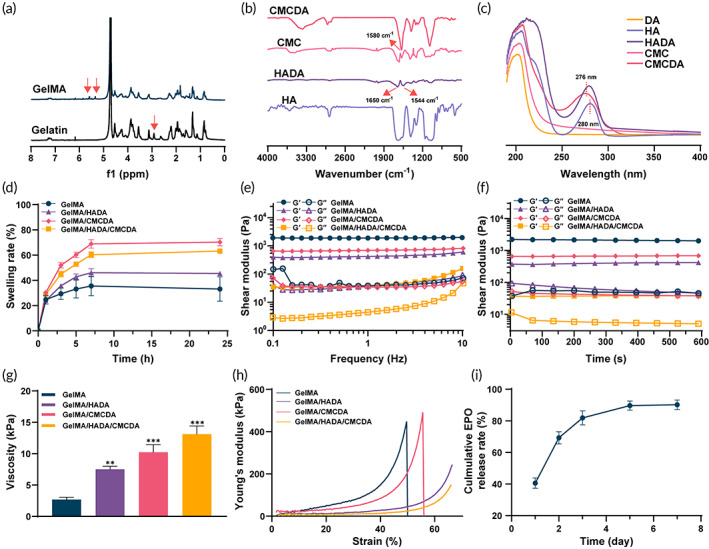
Preparation and characterization of the hydrogels. (a) ^1^H NMR spectra of Gel and GelMA. (b) FTIR spectra and (c) UV–vis absorption spectra of HA, HADA, CMC, and CMCDA. (d) Swelling capability assessment of hydrogels. (e) Frequency‐sweep test and (f) time‐sweep test of hydrogels. Note that G′ and G″ represented the elastic (storage) modulus and the viscous (loss) modulus, respectively. (g) The adhesive properties assessment of hydrogels to the porcine skins (*n* = 3). (h) Compressive curve of hydrogels. (i) The EPO release profile of GelMA/HADA/CMCDA/EPO hydrogels in 7 days. *p < 0.05, **p < 0.01, and ***p < 0.001

In this study, a novel GelMA/HADA/CMCDA hydrogel complex was prepared as a carrier for local ADSC implantation and sustained EPO release. The covalently crosslinked hydrogel was fabricated via photo‐initiated processes of GelMA. It is worth mentioning that gelatin is the hydrolysate of collagen, and only a small population of amino acid residues is involved in the process of MA modification, which maximizes the preservation of the functional motifs to imitate the component of ECM, such as RGD sequences for cell attachment and MMP sequences for cell remodeling.^31^ The presence of the acrylic protons (δ = 5.3 ppm and 5.5 ppm) in the product was determined by ^1^H‐NMR (Figure 1a). Meanwhile, to endow the biocompatible hydrogel with suitable adhesive property for cell localized implantation, DA grafted HA and CMC (HADA and CMCDA) were added to the system. HA and CMC are both derived from natural polysaccharide‐based biomaterials, which were reported to be helpful for inflammation inhibition, moisture retention, and cell migration.^33^, ^38^ The adhesive properties of polydopamine‐based hydrogels could be significantly improved for the reversible noncovalent bonds (π–π stacking and hydrogen bonding) mediated by the containing catechol groups. The DA modified products were confirmed by FTIR (Figure 1b) and UV–vis absorption spectra (Figure 1c). For FTIR, the presence of a peak at 1650 cm^−1^ belonging to the amide stretch vibration (*V*_C=O_) indicated the preparation of HADA, while the presence of a peak at 1580 cm^−1^ corresponding to the benzene ring skeleton vibration (*V*_C═C_) suggested the fabrication of CMCDA. Notably, the characteristic peak of the amide bond (*V*_C═O_) was not observed in the spectrum of CMCDA, which was considered to be masked by the C═C peak. For UV–vis absorption spectra, a slight blue shift (≈4 nm) was observed both in HADA and CMCDA, confirming the generation of amide linkages. Figure S1a showed the gelation process of GelMA/HADA/CMCDA hydrogels triggered by UV light. Figure S1b displayed the appearances of hydrogels with different components. Besides, the *SEM* results (Figure S1c) confirmed the interconnected and homogeneous pore structures of all hydrogels. In conclusion, these results confirmed the successful fabrication of polymers for further hydrogel preparation.

### 3.2 Physical and mechanical properties of hydrogels

The swelling ability was related to the porosity and the cross‐linking degree. Hydrogels with certain swelling abilities could prove good mechanical strength and water absorption for implanted cells for proliferation and nutrition.^39^ As shown in Figure 1d, hydrogels absorbed the PBS rapidly and reached their equilibrium after 7 h. Apparently, the supplement of HADA and/or CMCDA had improved the swelling performance of GelMA, which was attributed to the sponge‐like structure of CMC and the good water suction property of HA. The swelling ratio of GelMA and GelMA/HADA/CMCDA was 35.5% and 60.3%, respectively. Additionally, a decrease in swelling ratio was observed when HADA was added to GelMA/CMCDA, which might be ascribed to the enhanced crosslinking density.

We assumed that the introduction of HADA and CMCDA could significantly improve the mechanical strength of hydrogels for further implantation based on GelMA. To test this hypothesis, rheological analysis was first performed. As revealed in Figure 1e, the elastic modulus (G′) was significantly higher than the viscous modulus (G″), with shear frequency varying from 0.1 to 10 Hz among groups, indicating the solid elastic properties. As shown in Figure 1f, the time‐sweep test was also performed. Only a mild reduction was observed both in G′ and G″. Meanwhile, G′ was consistently higher than G″ throughout the experiment (600 s). The results suggested that hydrogels could maintain their structurally stable 3D network. Notably, GelMA/HADA/CMCDA demonstrated the lowest G′, indicating the most resilient performance.

Next, the adhesive properties and compression strength of hydrogels were evaluated. For adhesive properties assessment, hydrogels were applied between two pieces of fresh porcine skins and the lap shear tests were performed.^40^ As shown in Figure 1g, pure GelMA hydrogel displayed the lowest adhesive strength with approximately 3 kPa to porcine skin tissues. The supplement of HADA and/or CMCDA could significantly improve the adhesion strength of hydrogels as expected. The adhesive strength of GelMA/HADA/CMCDA had been enhanced three times than that of GelMA, which resulted from the reversible noncovalent bonds mediated by the containing catechol. Figure 1h exhibited the results of the compression test. Apparently, pure GelMA showed the strongest rigidity, with Young's modulus almost 400 kPa under a 50% compression strain. Compared to pure GelMA, hydrogels supplemented with HADA illustrated good resilient performance. GelMA/HADA and GelMA/HADA/CMCDA did not break at a high strain of 70%. Finally, the release behavior of EPO was determined by ELISA. Although catechol would react with the amine group of EPO, the number of amino groups on EPO was minimal, and the effect on hydrogels was negligible. As shown in Figure 1i, an initial burst effect was observed, and ≈70% EPO was released over 2 days, and the residual EPO was released slowly over the next few days. In the following research, we need to take measures to control the burst effect and achieve a more sustained and stable release of EPO. Considering the open wound in clinical practice, an ideal hydrogel with implanted stem cells for nED treatment should possess good mechanical properties to keep its integrity and good adhesive strength to ensure efficient topical transplantation of hydrogels in the nerve injury site. Herein, the prepared GelMA/HADA/CMCDA hydrogel was for suitable potential clinical application.

### 3.3 In vitro degradation assessment

As an implant material, it is essential for hydrogels to be biodegradable. Therefore, the degradation behavior of hydrogels was observed by immersing hydrogels in PBS. Lysozyme (1000 U/ml) was used to simulate the in vivo environment.^41^ The results are displayed in Figure S2. Obviously, the degradation rate of hydrogels was slower without lysozyme. It took about 14 days for hydrogels to degrade completely without lysozyme, while only about 7 days for hydrogels with lysozyme (Figure S2a,b). Additionally, compared with pure GelMA, hydrogels supplemented with HADA and/or CMCDA degraded faster in the first few days, since there was no chemical cross‐linking network between HADA/CMCDA and GelMA. *SEM* was also utilized to observe the changes in the microstructure of hydrogels (Figure S2c,d). Apparently, under the enzyme condition, the disintegration rate of the cavity in the hydrogel materials was faster than those without enzyme, which was consistent with the degradation profiles.

### 3.4 Biocompatibility of the hydrogels with ADSC

Compared with other kinds of stem cells, ADSC is easy to isolate from tissues and possesses the properties of higher activity to secrete neuroprotective related cytokines and immunoregulatory factors while lower immunogenicity.^34^, ^42^ As shown in Figure S3a1,2, ADSC displayed a fibroblast‐like morphology and spindle morphology. Also, ADSC has been widely developed in tissue engineering for its extensive differentiation potential. The positive Alizarin Red staining (Figure S3a3) and oil red O staining (Figure S3a4) confirmed its capacity to differentiate into osteocytes or adipocytes after specific induction. Besides, the obtained cells were identified by specific surface markers (Figure S3b), including negative expression of CD11b, CD45, and CD14, and positive expression of CD29, CD44, and CD90.

**FIGURE 2:**
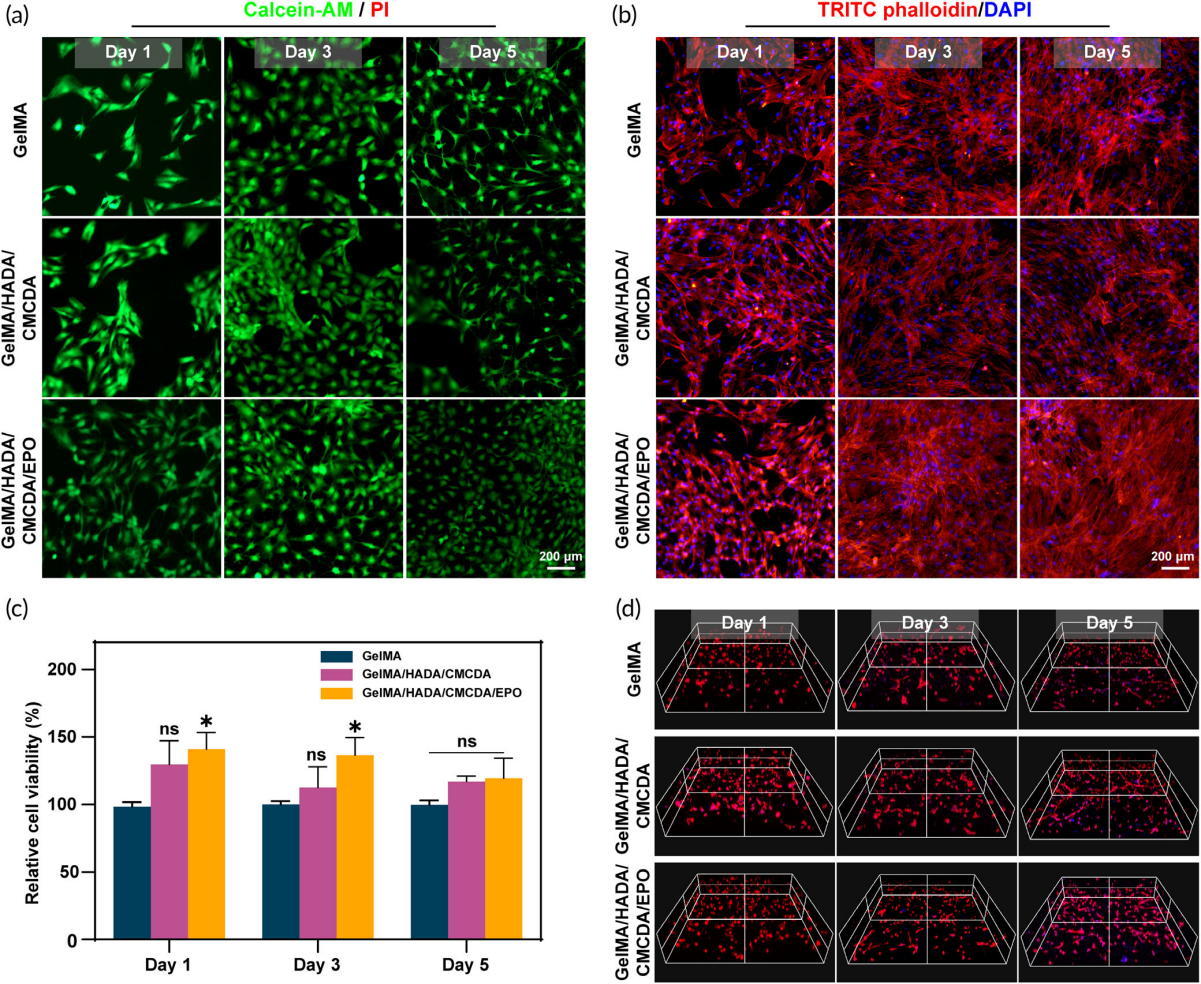
Effects of hydrogels on ADSC morphology, viability, proliferation, and spatial distribution. (a) Live/dead staining images of ADSC cultured on hydrogels at different time points. (b) Cell morphology of ADSC on hydrogels. (c) Cell viability of ADSC cultured on hydrogels by CCK‐8 assay. (d) Representative spatial distribution images of encapsulated cells inside hydrogels

Next, the effect of hydrogel materials on ADSC viability and proliferation was assessed. The survival of ADSC spreading on hydrogels was visualized via live/dead staining (Figure 2a). Little dead cells were detected in all co‐cultured hydrogel systems, indicating excellent biocompatibility. Meanwhile, Figure 2c showed the results of the CCK‐8 assay after 1, 3, and 5 days of co‐culture. No statistical significance was found in cell viability between GelMA and GelMA/HADA/CMCDA. To assess the optimum EPO concentration for treatment, Zhou et al.^28^ treated MSC with different concentrations of EPO (10, 100, 500, and 1000 IU/ml) and found that MSC treated with 500 IU/ml EPO displayed the highest proliferation rate and migration rate. Therefore, we selected 500 IU/ml as the experimental concentration. After the addition of EPO, the number of living cells increased. Previous studies have revealed that mesenchymal stem cells with pretreatment or the genetic modification of EPO could promote its proliferation and migration, which may be related to the activation of PI3K/Akt and ERK or inhibition of the P38MAPK pathway.^28^, ^43^ Similarly, the cytoskeleton morphology of ADSC was observed by TRITC Phalloidin staining of F‐actin. As shown in Figure 2b, most ADSC growing on GelMA/HADA/CMCDA/EPO hydrogel illustrated a more elongation, thicker, and more regular arranged filaments, which was important for cell migration.^28^, ^44^

### 3.5 ADSC encapsulation, viability, and spreading

The above results displayed the excellent biocompatibility and the potential of GelMA/HADA/CMCDA/EPO hydrogel as a carrier for cell implantation. Hence, we further analyzed the viability and spreading of ADSC in the 3D cell culture hydrogels. As shown in Figures 2d and S4a, ADSC was uniformly distributed in the 3D culture systems with a high survival rate. With the extension of co‐culture time, the OD values increased, indicating ADSC proliferation (Figure S4b). Notably, ADSC in GelMA/HADA/CMCDA/EPO hydrogel showed the highest OD value, suggesting the strongest proliferation capacity. As widely developed hydrogel materials, GelMA, HA, and CMC are important ECM components to enhance cell adhesion, viability, and proliferation.^31^, ^33^, ^38^ Herein, the prepared hydrogel was an ideal carrier for ADSC encapsulation and in vivo implantation.

### 3.6 The effects of hydrogels on the SC migration

**FIGURE 3:**
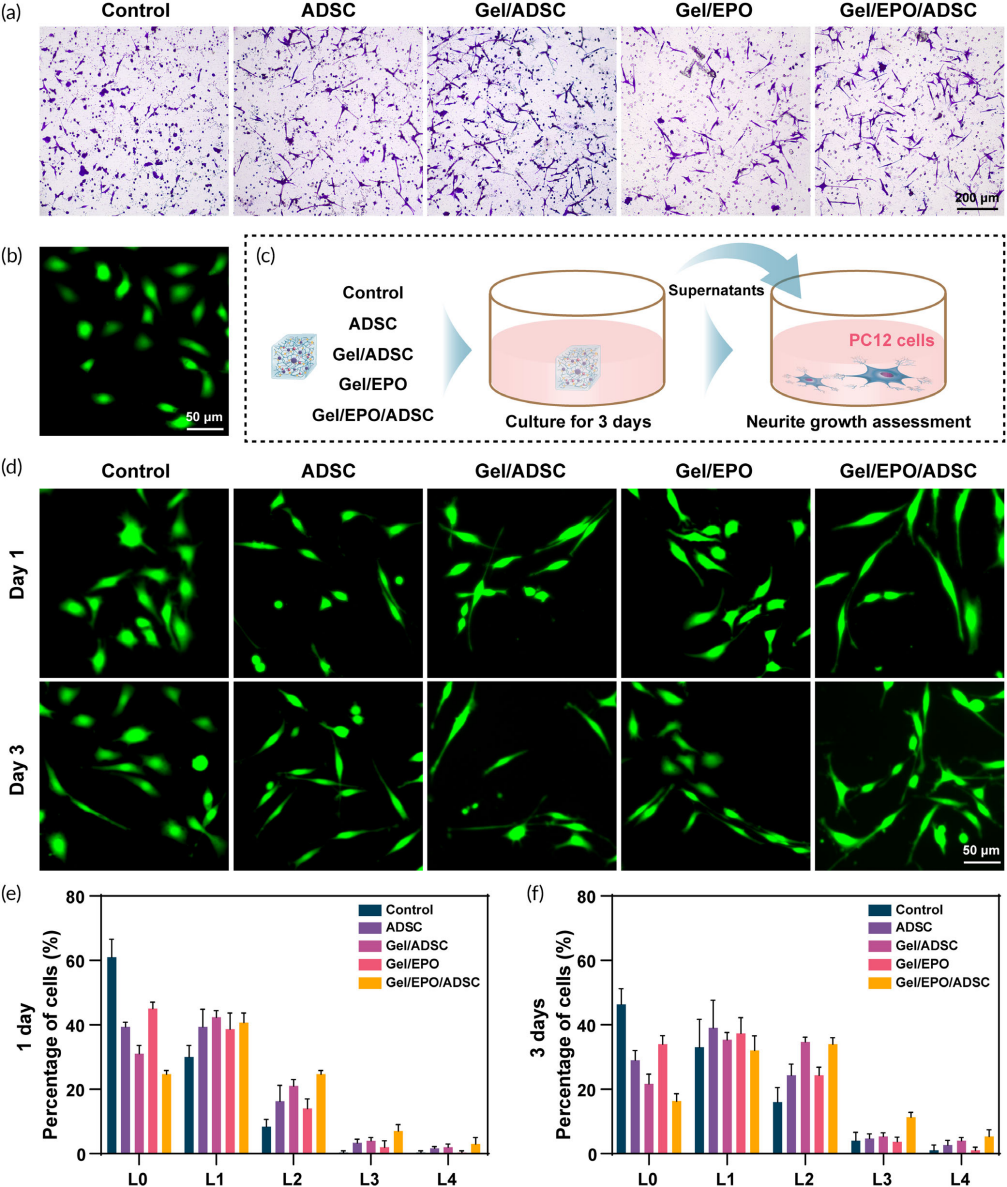
Migration of SC and differentiation promotion in PC12 cells among groups. (a) Captured images for migration of SC in response to different treatments. (b) Confocal microscopy images of poorly differentiated PC12 cells. (c) The supernatants of different cell co‐culture systems were harvest for the culture of PC12 cells. (d) Confocal microscopy images of Calcein‐AM‐labeled PC12 cells after different treatments. The level of growth was quantitatively divided into L0 to L4 according to the length of the neurites after 1 day (e) and 3 days (f) treatments. *p < 0.05 and **p < 0.01. Note that Gel represents GelMA/HADA/CMCDA hydrogel

Peripheral nerve regeneration is a complex biological process, and that immediately occurs after nerve injury. SCs are the primary cells that make up the peripheral nervous system, which is activated to phagocytize the debris in the initial time and enhance neurite growth by producing neuro‐protective related factors in the later period.^8^, ^45^ Considering the critical role of SC in nerve regeneration, we evaluated the effects of different hydrogel systems on cell migration. Primary SC isolated from the sciatic nerve were immunofluorescent identified by S100β^46^ (Figure S5a). Next, SC was incubated in the upper chambers. After being cultured for 48 h, the numbers of cells penetrating the lower chambers were evaluated. Figure 3a illustrated the representative results of each group, and Figure S5b was the semi‐quantitative analysis results. Obviously, the incubation of SC in ADSC supernatants led to more cell migration than the control group, and the chemotaxis effect was enhanced by seeding ADSC on hydrogels with/without the supplement of EPO. Interestingly, the supernatant of Gel/EPO alone could lead to certain numbers of cells penetrating the lower chambers, which was related to the effect of EPO on cellular migration.^28^, ^47^ To investigate the underlying mechanism of enhanced migration, the secretion levels of neuro‐protective related factors in supernatants were detected. As shown in Figure S6, the ADSC cultured on EPO‐loaded hydrogels showed the highest levels of GDNF, BDNF, NGF, and PDGFα proteins secretion, indicating the enhanced paracrine activity.

### 3.7 Neurite growth assessment on PC12 cells

To verify the capability of the hydrogel complex in nerve regeneration, PC12 cells were incubated with the supernatants, and the morphology was observed to evaluate the effects of hydrogels on cell differentiation (Figure 3c). As shown in Figure 3b, undifferentiated PC12 cells presented the common spindle‐shaped or polygonal shapes. Figure 3d illustrates the representation images after incubation for 1 day and 3 days. Compared with the control group, cells cultured in supernatants presented typical nerve cell morphology, with some multipolar cells with longer synapses. We further divided differentiated PC12 cells into five groups (from L0 to L4) to quantify the elongation of neural processes^36^ (Figure 3e,f). Enhanced cell differentiation was observed even within a 1‐day treatment. The percentage of L0 was about 60% in the control group versus 30% in Gel/EPO/ADSC group. At the end of the third day, the percentage of L0 in control groups remained about 47%, while the rate decreased to about 17% in Gel/EPO/ADSC group, with an increased percentage of highly differentiated cells (from L2 to L4). Additionally, PC12 cells in other treatment groups also showed varying degrees of differentiation, suggesting that the capacity in nerve repair of ADSC was enhanced by culturing on hydrogels. Meanwhile, the cell differentiation appeared to be mildly improved compared with the control group, which was ascribed to the increased mitochondrial activity and the attenuation of oxidative stress effect of EPO on PC12 cells.^48^ Together, these results strongly verified the potential of stem cell‐EPO‐hydrogel complex in the promotion of neurite outgrowth.

### 3.8 Erectile function restoration in BCNI rats

**FIGURE 4:**
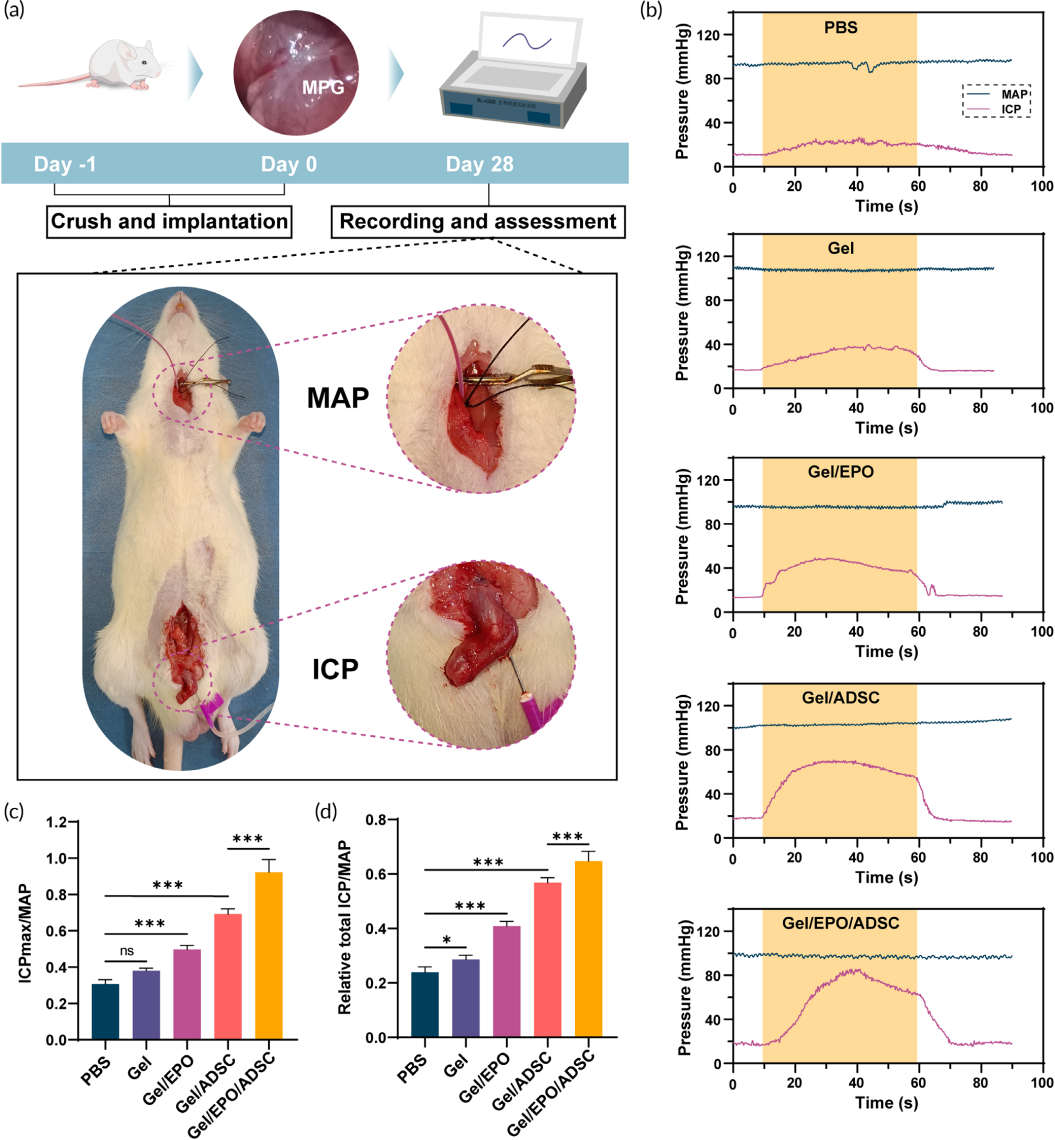
Erectile function restoration of rats after treatments of the hydrogel complex. (a) Schematic illustration of the experiment and the measurement method of ICP and MAP. (b) Under 5‐V electrical stimulus to cavernous nerve, changes of ICP and MAP were recorded among the five groups. (c) Maximum ICP (ICPmax)/MAP values and (d) relative total ICP/MAP values in the five groups after 4 weeks of implantation (*n* = 5). One‐way ANOVA followed by post hoc Bonferroni analysis. *p < 0.05 and ***p < 0.001. Note that Gel represents GelMA/HADA/CMCDA hydrogel

Encouraged by the above enhancement of nerve regeneration, the effects of EPO‐loaded GelMA/HADA/CMCDA hydrogel on erectile function improvement and synergism with ADSC were assessed via local implantation in a rat BCNI model. The EPO‐loaded GelMA/HADA/CMCDA hydrogel encapsulated with ADSC (Gel/EPO/ADSC group) was immediately injected to the local nerve crush site and polymerized by UV irradiation, with merely PBS implantation (PBS group), GelMA/HADA/CMCDA hydrogel implantation (Gel group), EPO‐loaded GelMA/HADA/CMCDA hydrogel implantation (Gel/EPO group), and GelMA/HADA/CMCDA hydrogel encapsulated with ADSC implantation (Gel/ADSC group) performed in parallel as controls (Figure 4a). Four weeks after treatment, the MAP and ICP were recorded to reflect the erectile process in each group. Under the same electrical stimulus to the cavernous nerve, the values of MAP and ICP were recorded, and the ratio of ICP_max_ to MAP and the ratio of relative total ICP (area under the curve) to MAP were calculated to evaluate the erectile functions. Generally, no difference was found in MAP among groups. After the crush to CN, rats exhibited the most severe ED (PBS group), while varying degrees of function improvement was achieved after the treatment of different hydrogel complex, with the most effective therapy in the Gel/EPO/ADSC group (Figure 4b–d). The strategy of EPO‐loaded hydrogel could significantly enhance the therapeutic effect of ADSC (*p* < 0.001). In addition, EPO receptor expression was identified and localized in penile tissues and the periprostatic neurovascular bundles responsible for erectile function.^30^ Allaf et al.^49^ reported that exogenous administration of 5000 IU/kg in the setting of BCNI promoted erectile function recovery. In our study, the ICP_max_/MAP value and relative total ICP/MAP value of Gel and Gel/EPO group were higher than those of the PBS group, suggesting the benefits of hydrogel materials and EPO in function restoration.

### 3.9 Cell tracing

At present, stem cells have been preliminarily explored in nED treatment research, of which the administration routes are IC and IP.^50^, ^51^ It has been reported that the route of IC focuses on nerve regeneration, while that of IP attenuates the degeneration of penile tissues caused by neuronal Wallerian degeneration.^52^ Although the two approaches showed similar therapeutic effects in short‐term treatment (2 or 4 weeks), as a neurotraumatic disease, giving priority to rapid repair of damaged nerves is the best choice to deal with nED. Fandel et al.^37^ observed that the early migration of stem cells toward the MPG/CN accounted for the therapeutic effect via IC. Meanwhile, although cell‐fibrin scaffolds were usually adopted to encapsulate stem cells in the administration route of IC, the therapeutic efficacy was severely compromised by the poor accumulation in the injury site to function effectively. It, therefore, seems that the implantation of abundant cells with high viability could lead to improved performance of stem cells in the restoration of erectile function. In this study, we proposed an innovative method for accurate delivery of ADSC to the injury site via an adhesive hydrogel. First, we assessed the adhesive property of hydrogels in vivo. After treatment with hydrogels for 1, 3, and 7 days, rats were anesthetized and dissected. As shown in Figure S7a, the residual hydrogels were found around the periprostatic area, indicating the good adhesive property of hydrogels for efficient retention at the injured area for a relatively long time. These results were consistent as expected and met the prerequisite for cell implantation in vivo.

Next, the residual hydrogels were harvested and observed under a fluorescence microscope. Interestingly, a large amount of PKH26 labeled ADSC was detected on the first day, and only a small number of labeled cells remained on the 7th day (Figure S7b). Compared with the results previously reported that stem cells were only detectable in the first 2 days after IP,^18^ it could be inferred that the EPO‐loaded hydrogel provided a shelter for ADSC from the complicated in vivo environment, compromising for excessive cell loss and death. Meanwhile, it was not difficult to find that the density of PKH26‐labeled cells decreased significantly on the 7th day than that on the first day, which was ascribed to the degradation of the hydrogel.

To further investigate the fate of the delivered ADSC, the MPG tissues were collected and sliced for the monitor of PKH26 labeled ADSC at 3 d, 7 d, and 14 d. As shown in Figure S7c, only a few ADSC were detectable in MPG tissues after implantation, suggesting that the therapeutic effect of ADSC was not exerted by entry to nerve tissues and differentiation into neural cells. At present, the multipotency of differentiation into specialized cell types and the paracrine effect are the two main mechanisms for the therapeutic effects exerted by stem cells. Previous studies suggested that stem cells restored erectile function in BCNI rats by activating the host's prolonged secretion of many critical soluble factors.^15^ Herein, we found that only a small number of stem cells migrated into the tissues, but did not explore whether stem cells differentiate into other types of cells. Based on experiments in vitro and previous reports, paracrine seems to be the main mechanism for stem cells in nED treatment.

### 3.10 Nerve regeneration assessment

**FIGURE 5:**
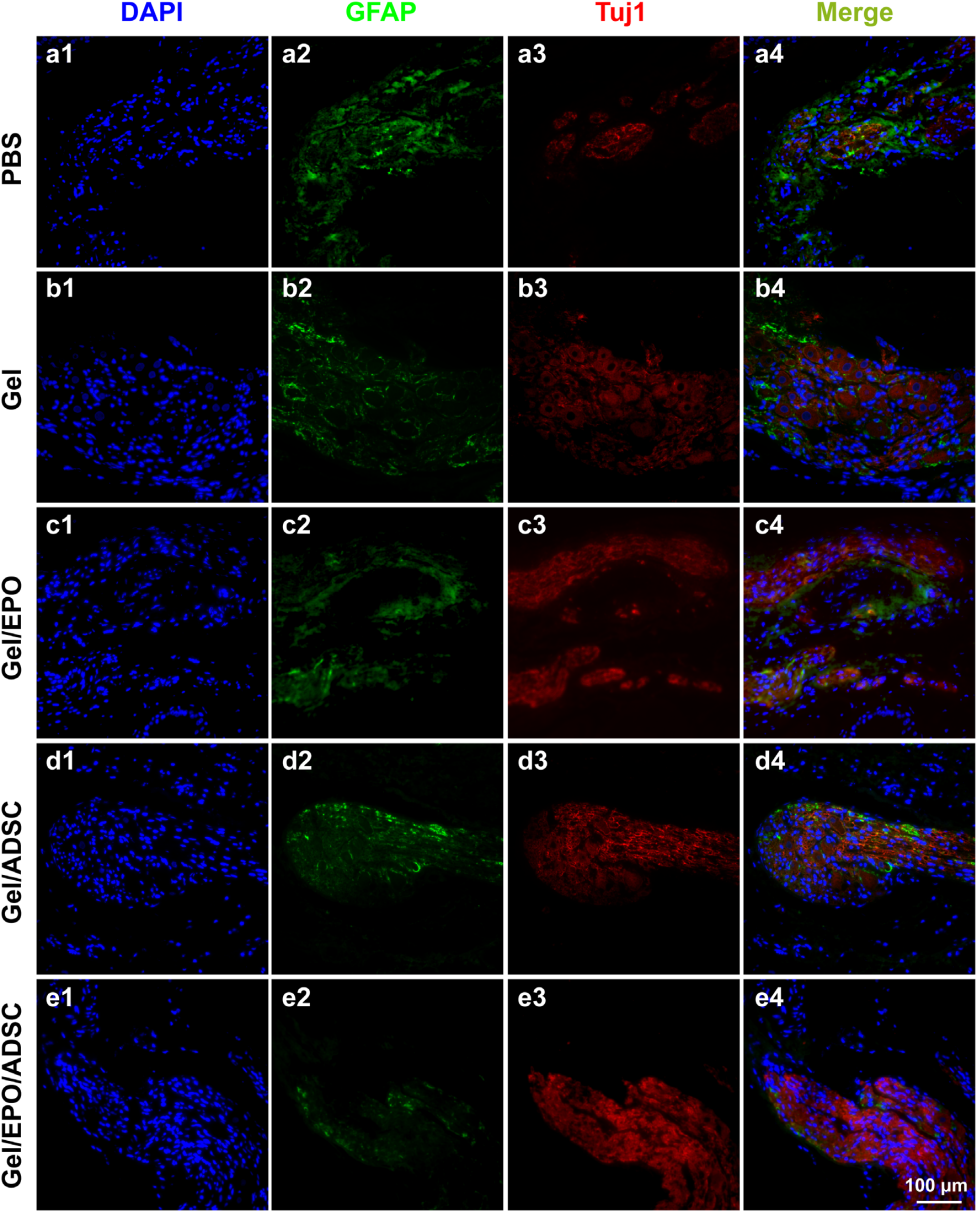
GFAP‐positive cells (for astrocytes, green) and Tuj1‐positive cells (for neurons, red) of MPG/CN on Day 28 among groups

Regeneration of an injured nerve is a complex biological process involving the infiltration of neurons and myelination, the chief obstacle of which is glial scar formation. To further understand the underlying mechanism of the erectile function restoration, immunofluorescence staining of MPG/CN was carried out to observe the nerve regeneration elicited among groups. Herein, the expression level of class III β‐tubulin (Tuj1) for neuronal cells and glial fibrillary acidic protein (GFAP) for activated astrocytes were detected, respectively. Fluorescence microscopy images demonstrated significant differences in the density and distribution of Tuj1 and GFAP among groups (Figure 5). The expression level of GFAP was significantly greater in the PBS group in comparison with treatment groups, indicating the therapeutic effects in inhibiting the formation of a glial scar after nerve injury. Meanwhile, the Tuj1 immunostaining level demonstrated a negative correlation with GFAP, suggesting nerve regeneration after hydrogels implantation (Figure S8). Thus, hydrogel complex had greatly improved neuronal content and inhibited astrocytic response at the same time in BCNI rats. In addition, among groups, the EPO‐loaded hydrogel synergizing with ADSC demonstrated the most effective therapy. It could be inferred that the EPO‐hydrogel‐ADSC strategy was able to promote nerve regeneration while inhibiting glial scar formation, leading to an improved functional restoration performance.

### 3.11 Rehabilitation of target penile tissues

**FIGURE 6:**
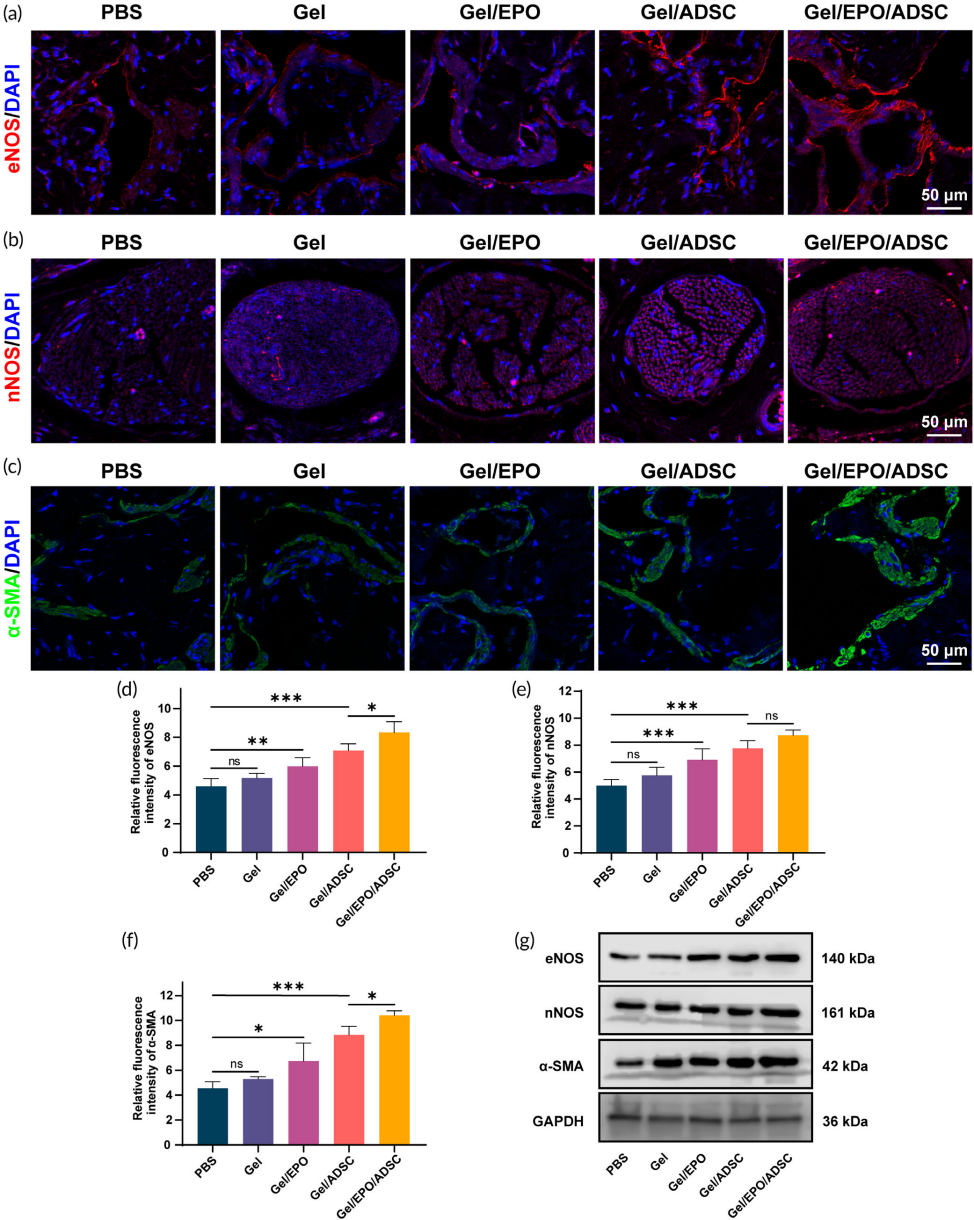
Implantation of EPO‐loaded hydrogel encapsulated with ADSC improved rehabilitation of target penile tissues. Fluorescent immunostaining of eNOS (a, d), nNOS (b, e), and α‐SMA (c, f) in the corpus cavernosum. (g) Representative images of western blot analysis for eNOS, nNOS, and α‐SMA in the corpus cavernosum. One‐way ANOVA followed by post hoc Bonferroni analysis. *p < 0.05 and ***p < 0.001. Note that Gel represents GelMA/HADA/CMCDA hydrogel

A good neural network, an intact vascular system, and healthy cavernosal tissue ensure sufficient penile erection. Damage to CN will lead to a series of pathophysiological changes in the architecture of the penis, such as loss of endothelial tissues, denervation, and decrease of cavernosal smooth muscle ingredient.^53^, ^54^ Moreover, these changes were reported to continue to progress in the later period, even if CN was successfully repaired by that time.^55^ To further observe the rehabilitation of the penile tissues elicited by different treatments, immunofluorescent staining of penis transverse sections was performed. Endothelial tissue is the guarantee of the sufficient engorgement of the penis during erections, of which the recognized marker is eNOS.^56^ As shown in Figure 6a,d, treatments with Gel/EPO, Gel/ADSC, and Gel/EPO/ADSC could restore eNOS expression in different degrees, suggesting the rehabilitation of endothelial components. NO‐cGMP is a vital pathway to mediate penile erection, with nNOS (neuronal nitric oxide synthase) as a vital molecule.^56^ The expression level of nNOS in the dorsal penile nerve was able to reflect the response‐ability of the penis to neurotransmitter (nitric oxide) signals. Apparently, BCNI led to significant downregulation of nNOS (PBS group), which could be rescued in hydrogel treatment groups (Figure 6b,e). Gel/ADSC and Gel/EPO/ADSC group displayed the strongest and no significantly different therapeutic effect. Corpus cavernosum smooth muscle (CCSM), the structural basis of cavernous space relaxing and penile erection, has a vital role in changing hemodynamics during penile erection.^57^ Previous studies revealed that CCSM undergoes a shift in phenotypes from a contractile state to a synthetic state in a rat model of BCNI.^58^ Therefore, the expression level of α‐SMA, an excellent contractile marker, was also evaluated to observe the smooth muscle content. Compared with the PBS group, treatment groups with hydrogels, especially the Gel/EPO/ADSC hydrogel, illustrated a stronger fluorescence intensity of α‐SMA, indicating the restoration of smooth muscle ingredient (Figure 6c,f). Notably, the smooth muscle in Gel/EPO/ADSC group was more intact than other groups. In addition, a similar trend in the protein levels of eNOS, nNOS, and α‐SMA in penile tissues was confirmed by western blot analysis (Figure 6g).

Taken together, these results showed consistent outcomes with erectile function detection and nerve regeneration assessment, suggesting that the EPO‐loaded hydrogel synergizing with ADSC contributed to restoring nED via enhancing rapid nerve regeneration and penile rehabilitation.

### 3.12 Apoptosis evaluation

**FIGURE 7:**
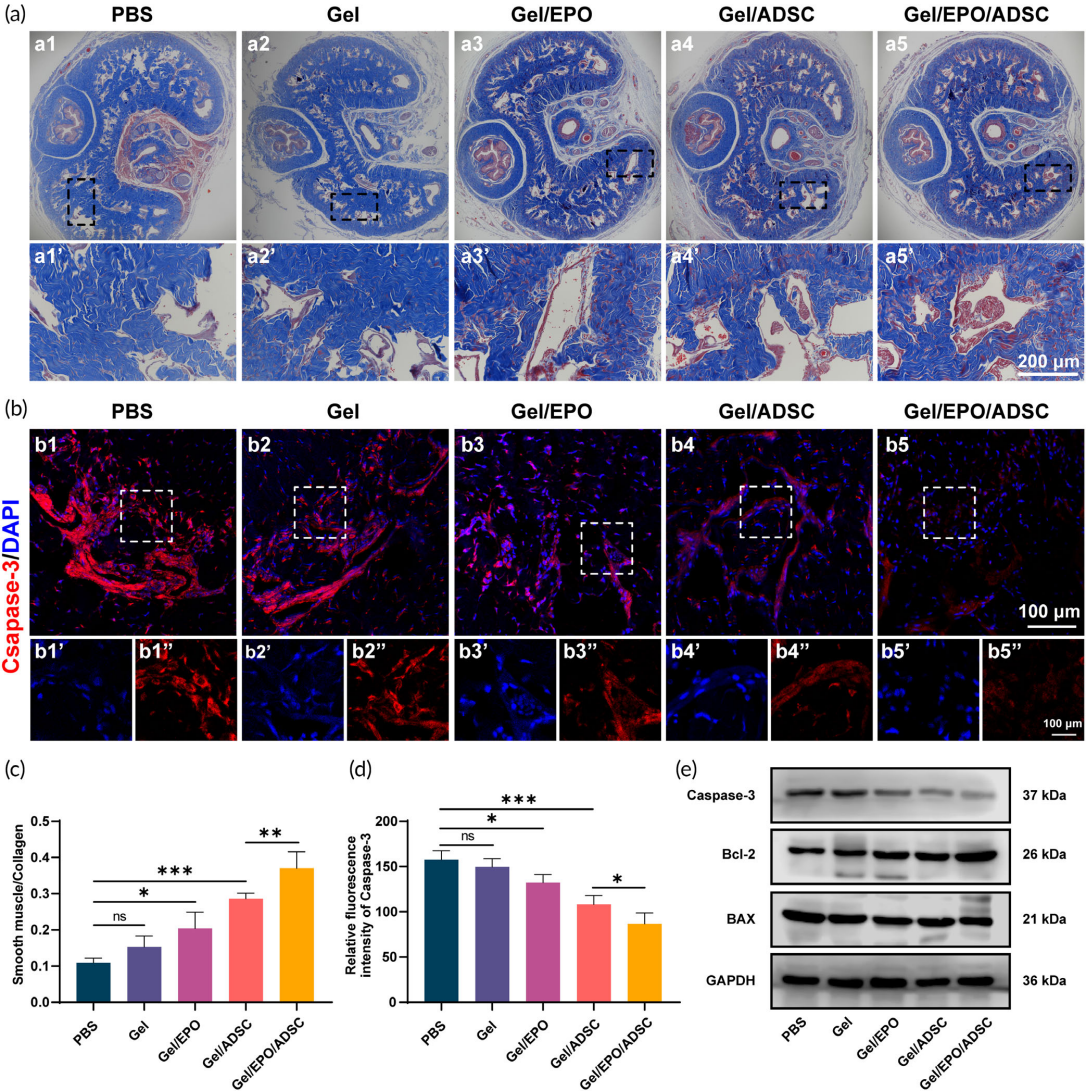
ADSC‐EPO‐hydrogel combing strategy preserved vascular endothelium content and inhibited penile fibrosis via exerting anti‐apoptotic effects. (a) Masson's trichrome staining of the penile and the smooth muscle/collagen ratio (c) among groups. Fluorescent immunostaining (b) and relative fluorescence intensity of Caspase‐3 (d) among groups. (e) Caspase‐3, Bcl‐2, and BAX protein expression in penile tissues. One‐way ANOVA followed by post hoc Bonferroni analysis. *p < 0.05, **p < 0.01, and ***p < 0.001. Note that Gel represents GelMA/HADA/CMCDA hydrogel

Nerve injury can lead to different degrees of dysfunction of target organs. Although the damage is limited to the neuron, the functional recovery involves the neuronal cell body, axon, and target organ. Therefore, the study of nerve injury mainly includes three aspects: the protection of neurons, the promotion of nerve regeneration, and the prevention and treatment of denervated target organ atrophy. The delay or irreversible damage of any of these links will affect the rehabilitation of function, ultimately affecting the curative effect. Apoptosis and fibrosis are essential mechanisms leading to nED, which was reported to occur as early as 1 week after BCNI.^34^, ^54^ It is therefore necessary to repair the nerve quickly and effectively in the early stage. In this study, the appropriate degradation rate and adhesive properties of hydrogels, the sustained release of EPO, and the enhanced viability of ADSC were well satisfied with the critical time window of nED treatment. Herein, to assess whether the curative effect of nerve regeneration could alleviate apoptosis and fibrosis of penile tissues (target organ), Masson trichrome staining of the corpus cavernosum was first performed. Figure 7a shown the representative results of each group, in which smooth muscle was stained red while connective tissue was stained blue, respectively. Obviously, BCNI led to severe atrophy of cavernosa smooth muscle, with the lowest ratio value of smooth muscle to collagen (PBS group). Meanwhile, treatment with ADSC could restore the smooth muscle ingredient in penile, of which the curative effect was enhanced by the EPO‐loaded hydrogel implanted strategy (Figure 7c). Similar curative results were observed in immunofluorescent staining of caspase‐3 on penile tissues (Figure 7b,d). In addition, the expression levels of apoptosis‐related proteins (caspase‐3, Bcl‐2, and BAX) in penile tissues was explored via western blot analysis (Figure 7e). After an injury to MPG/CN, the expression levels of caspase‐3 and BAX were increased while that of Bcl‐2 was decreased, indicating the process of apoptosis. The implantation of ADSC could attenuate these effects. Thus, it could be inferred that the stem cell‐EPO‐hydrogel implanted strategy could prevent the atrophy of the penis via inhibiting apoptosis and the fibrosis process.

## 4 CONCLUSION

In this study, we designed and fabricated an EPO‐loaded multifunctional hydrogel encapsulated with ADSC for the effective restoration of nED. The improved adhesive property and mechanical strength of hydrogels were achieved by introducing catechol‐catechol adducts HADA and CMCDA, thereby effectively ensuring ADSC accumulation in the injury site. Meanwhile, the hydrogel sustaining release of EPO improved the viability of ADSC, leading to enhanced proliferation and paracrine activity of ADSC. In vitro results showed that the multifunctional hydrogels could promote the migration of SCs and the differentiation of PC 12 cells. On a bilateral cavernous nerve injury rat model, the stem cell‐EPO‐hydrogel combing strategy induced a significant erectile function restoration. Meanwhile, the hydrogels could promote nerve regeneration while inhibiting glial scar formation. More importantly, timely and effective nerve repair could inhibit apoptosis and enhance the rehabilitation of the denervated penile tissues. Our results, collectively, present a promising strategy not only for the restoration of neurogenic erectile function but also for the clinical translation of stem cell therapy.

## AUTHOR CONTRIBUTIONS

**Jun Shao:** Conceptualization (lead); formal analysis (lead); investigation (lead); methodology (lead); validation (equal); writing – original draft (lead); writing – review and editing (equal). **Pan Nie:** Data curation (equal); investigation (equal); methodology (lead). **Wende Yang:** Conceptualization (equal); investigation (equal); methodology (equal). **Rui Guo:** Conceptualization (equal); methodology (lead); resources (lead). **Dongbing Ding:** Data curation (equal); formal analysis (equal); validation (equal). **Rongpu Liang:** Investigation (equal); writing – original draft (supporting). **Bo Wei:** Conceptualization (equal); funding acquisition (equal); supervision (equal); validation (equal). **Hongbo Wei:** Conceptualization (equal); funding acquisition (equal); project administration (equal); supervision (equal); validation (equal).

## CONFLICT OF INTERESTS

The authors declare no conflict of interests.

### PEER REVIEW

The peer review history for this article is available at https://publons.com/publon/10.1002/btm2.10319.

## Supporting information


**DATA S1** Supplementary Figures

## Data Availability

The data that support the findings of this study are available from the corresponding author upon reasonable request.
