# Supplementary material for: An EPO‐loaded multifunctional hydrogel synergizing with adipose‐derived stem cells restores neurogenic erectile function via enhancing nerve regeneration and penile rehabilitation
Source: Bioeng Transl Med. 2022 May 31;7(3):e10319. doi: 10.1002/btm2.10319 (PMC9471998; doi:10.1002/btm2.10319)
Supplement: Supplementary file 1 — DATA S1 Supplementary Figures [file BTM2-7-e10319-s001.docx]

**Supporting information**

An EPO-loaded multifunctional hydrogel synergizing with **Adipose-derived stem cells (ADSC) restores neurogenic erectile dysfunction** via enhancing nerve regeneration and penile rehabilitation

Jun shao^a^, Pan Nie^a^, Wende Yang^a^, Rui Guo^b^, Dongbing Ding^a^, Rongpu Liang^a^, Bo Wei^a,*^ , Hongbo Wei^a,**^

^a^ Department of Gastrointestinal Surgery, the Third Affiliated Hospital of Sun Yat-sen University, Guangzhou 510630, China

^b^ Key Laboratory of Biomaterials of Guangdong Higher Education Institutes, Guangdong Provincial Engineering and Technological Research Centre for Drug Carrier Development, Department of Biomedical Engineering, Jinan University, Guangzhou 510632, China

^*^ Corresponding author.

^**^ Corresponding author.

E-mail address: [weibo3@mail.sysu.edu.cn](mailto:weibo3@mail.sysu.edu.cn) (B. Wei), [weihb@mail.sysu.edu.cn](mailto:weihb@mail.sysu.edu.cn) (H. Wei)


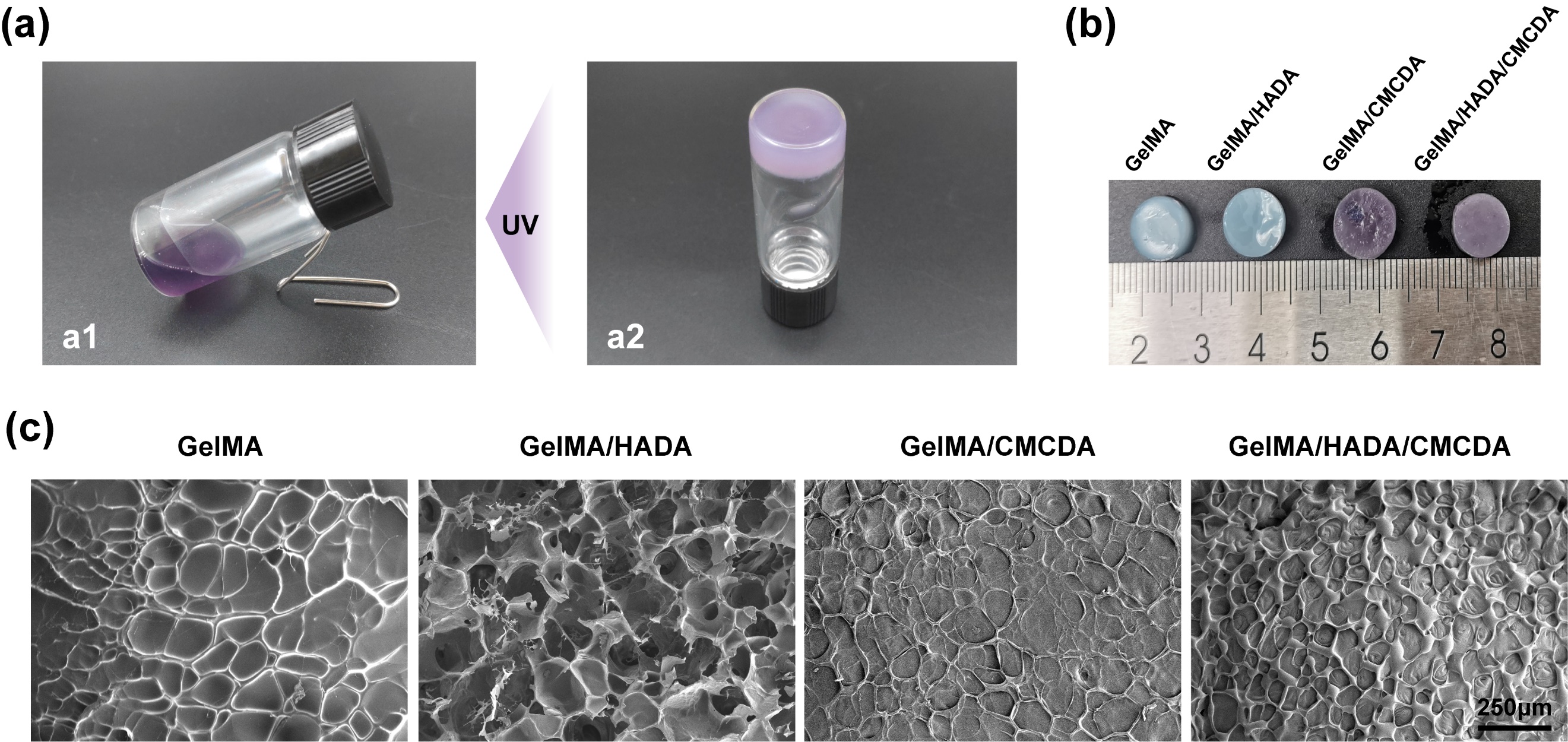


Figure S1. (a) The process of gelation before (a1) and after (a2) a UV crosslinking. (b) The appearances of cylindrical hydrogels with different components. (c) SEM micrographs of hydrogels with different components showing highly porous 3D structures.


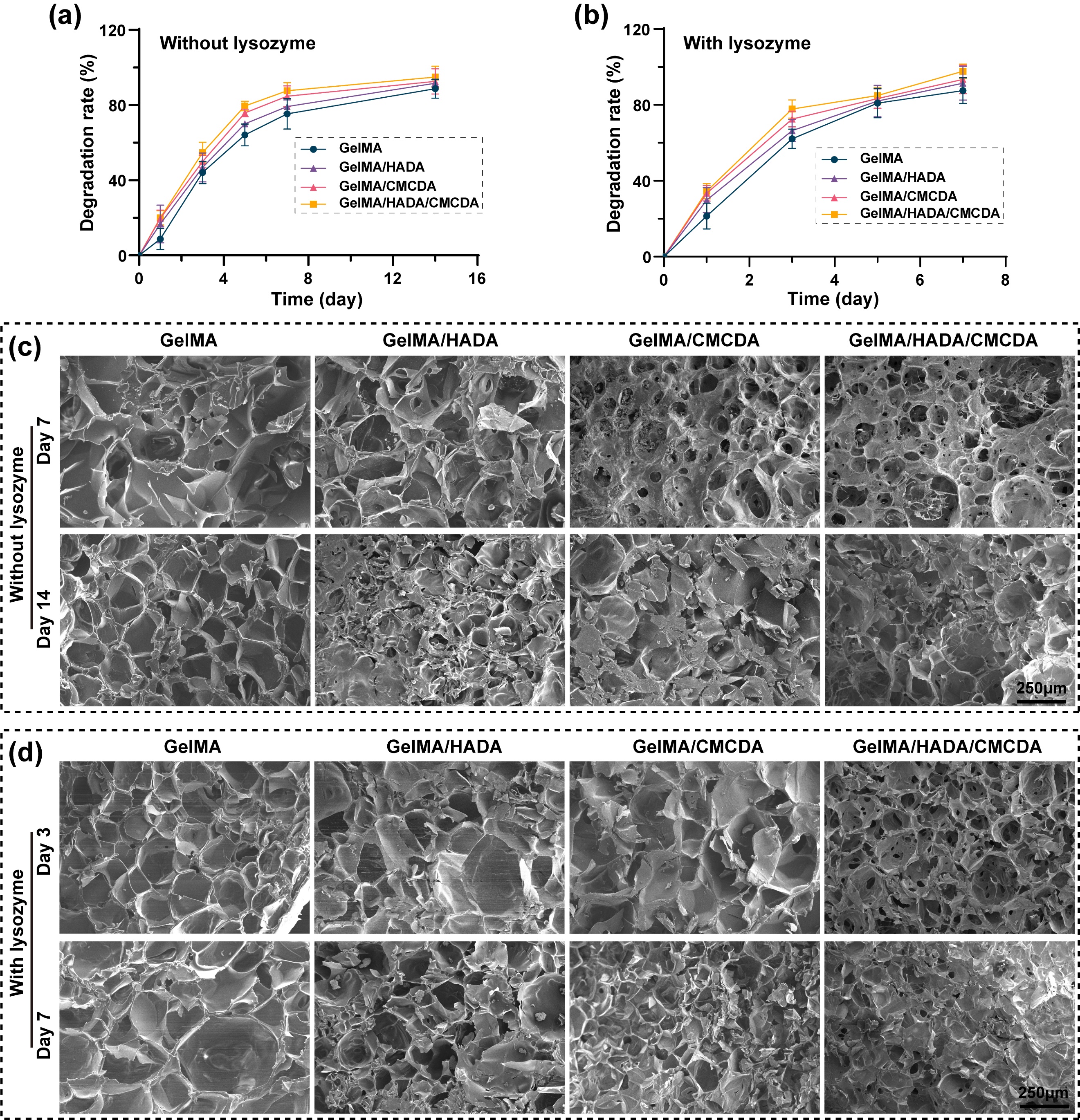


**Figure S2.** The degradation rate of hydrogels in PBS with (a) or without (b) the supplement of lysozyme at indicated time points. SEM images of degrading hydrogels with (c) or without (d) the supplement of lysozyme at indicated time points.


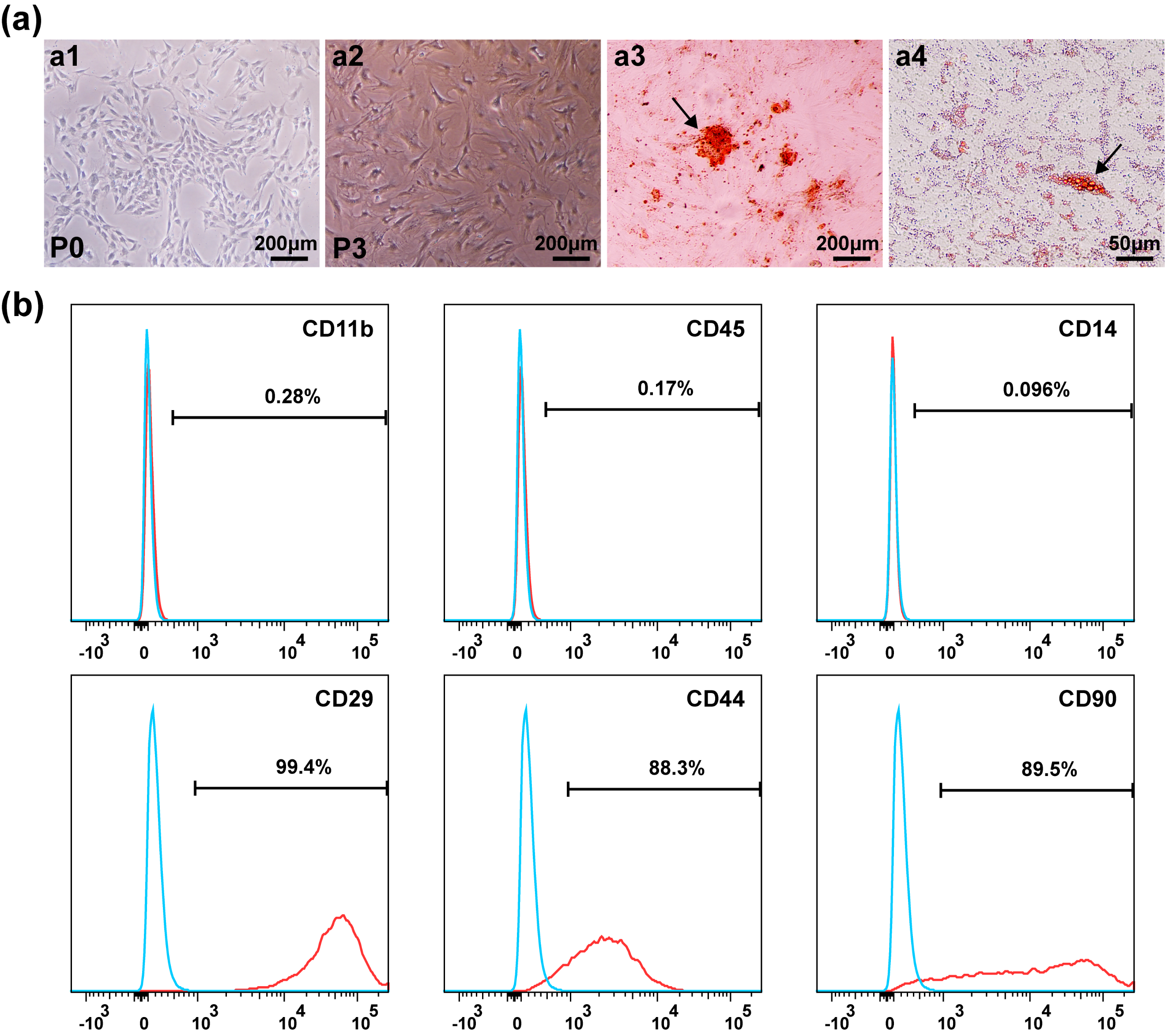


**Figure S3.** Isolation and characterization of ADSC. Morphology of ADSC in passage 0 (a1) and passage 3 (a2). after induction, ADSC demonstrated the typical phenotype of osteocytes (a3) or he typical phenotype of adipocytes (a4). (b) Flow cytometric analysis of surface markers showed that ADSC expressed less endothelial or hematopoietic markers CD11b, CD45, and CD14, but more stem cell markers, including CD29, CD44, and CD90.


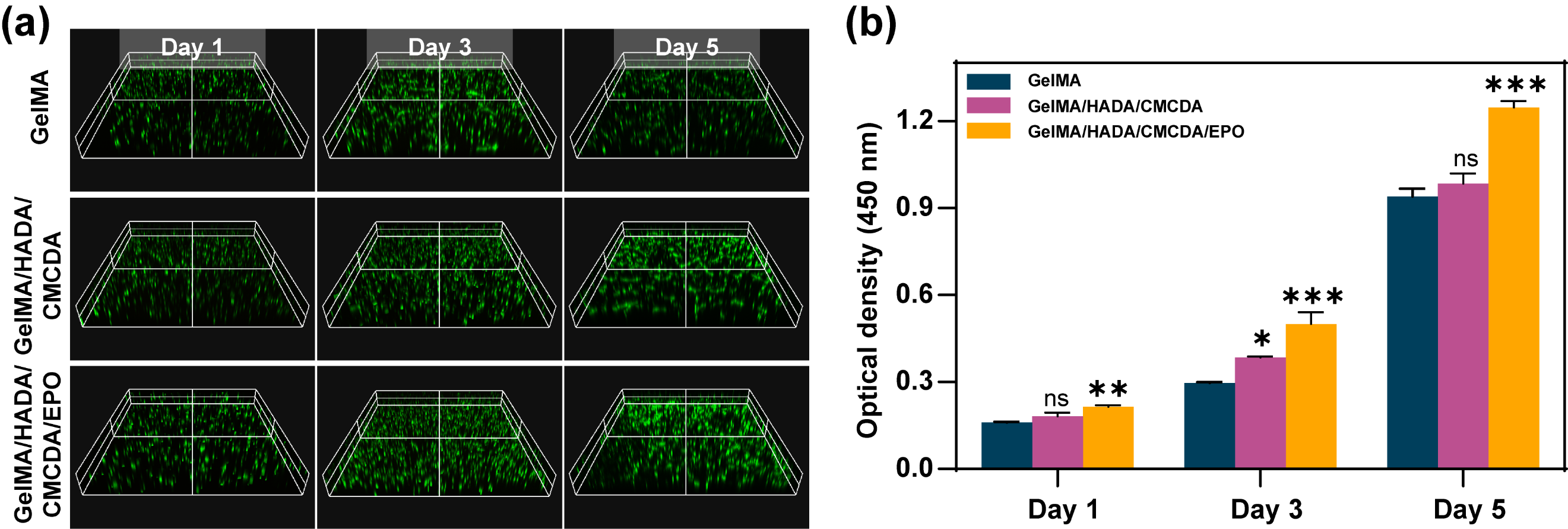


**Figure S4.** (a) Representative 3D images of encapsulated cells inside hydrogels. (b) Proliferation of encapsulated ADSC inside hydrogels by CCK-8 assay. (*) P < 0.05, (**) P < 0.01, and (***) P < 0.001.


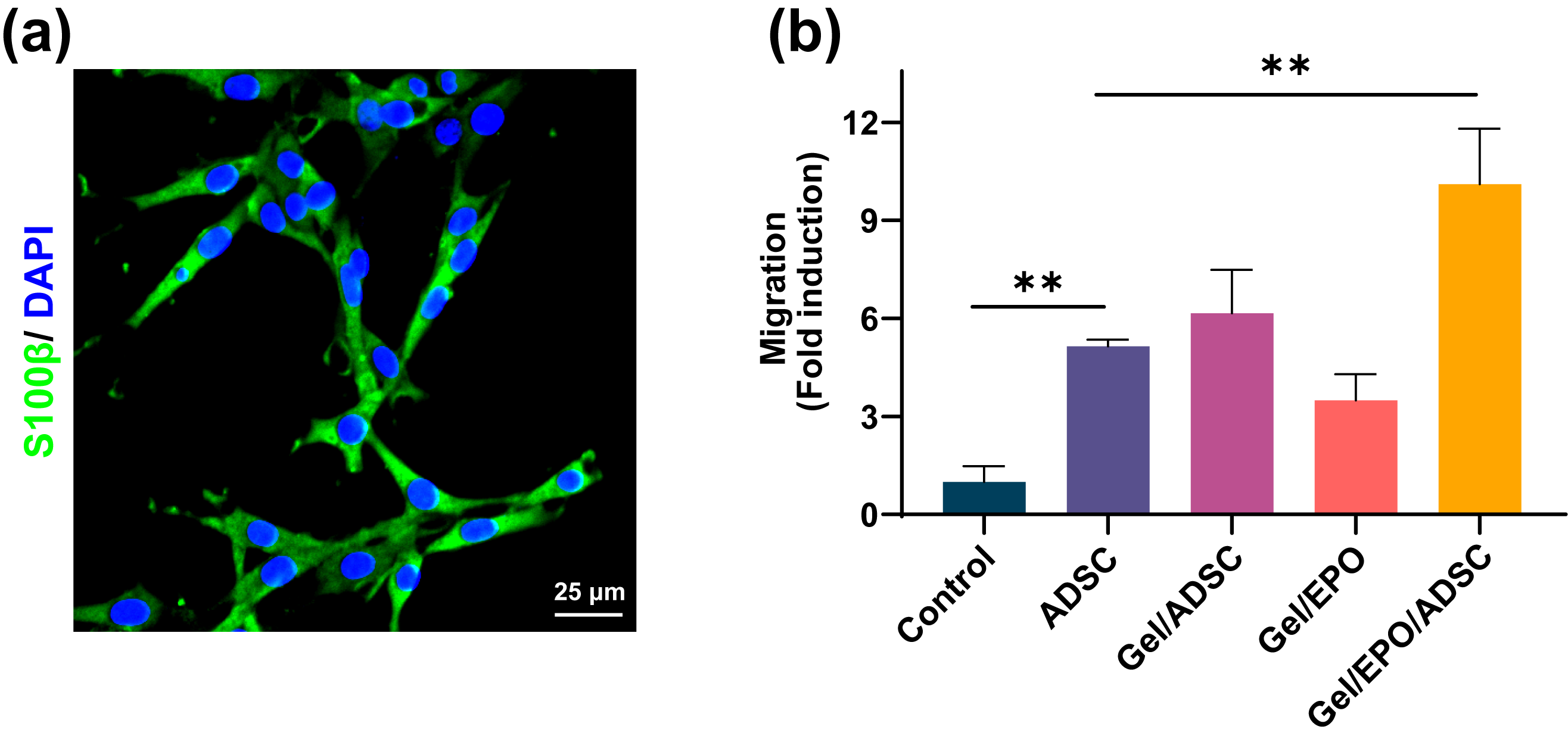


Figure S5. (a) identification of primary SC. (b) Quantification of migrated SC. (**) P < 0.01. Note that Gel represents GelMA/HADA/CMCDA hydrogel.


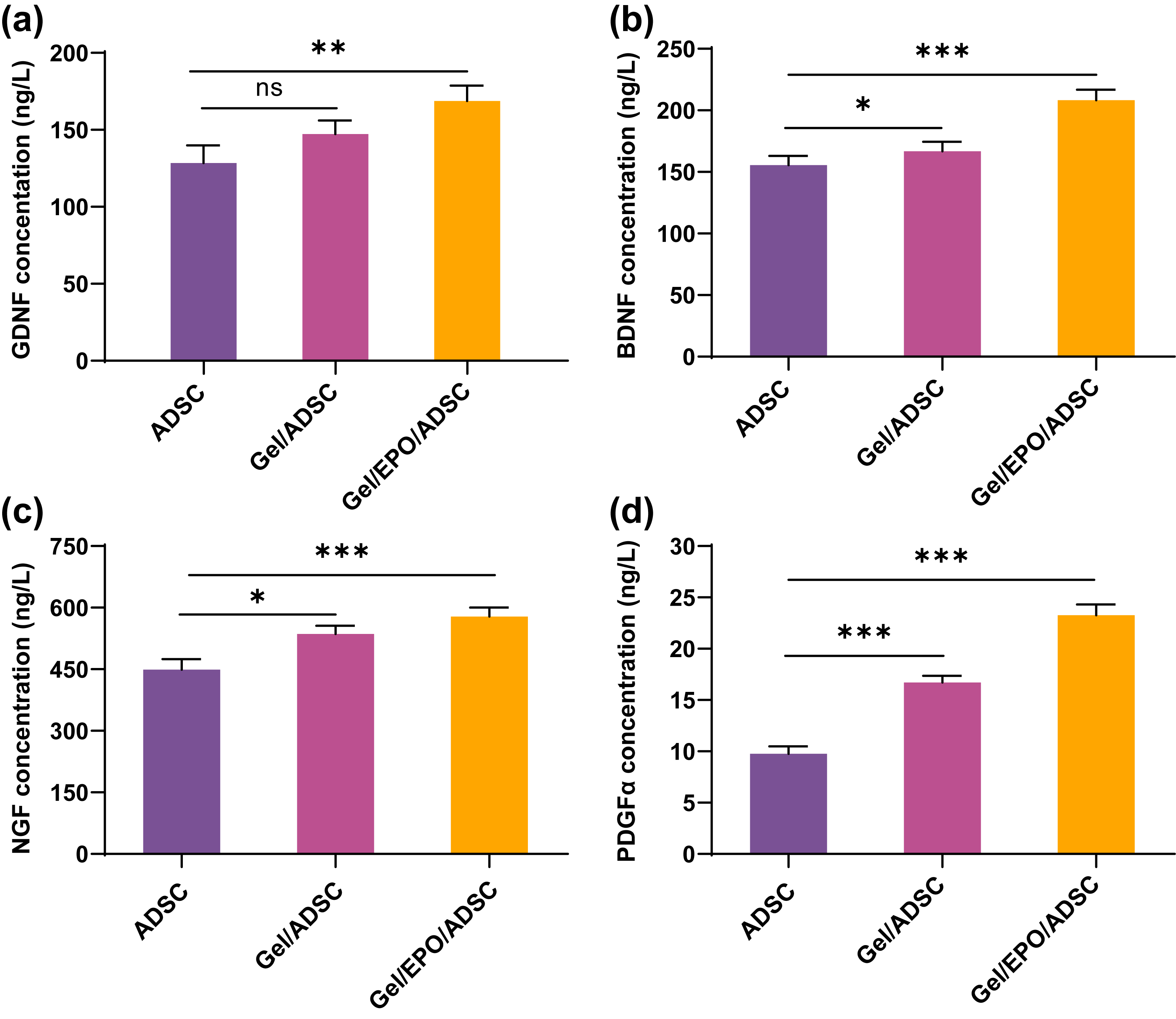


**Figure S6.** ELISA assay results for detection of the (a) GDNF, (b) BDNF, (c) NGF, and (d) PDGFα proteins concentration among groups. (*) P < 0.05, (**) P < 0.01, and (***) P < 0.001. Note that Gel represents GelMA/HADA/CMCDA hydrogel.


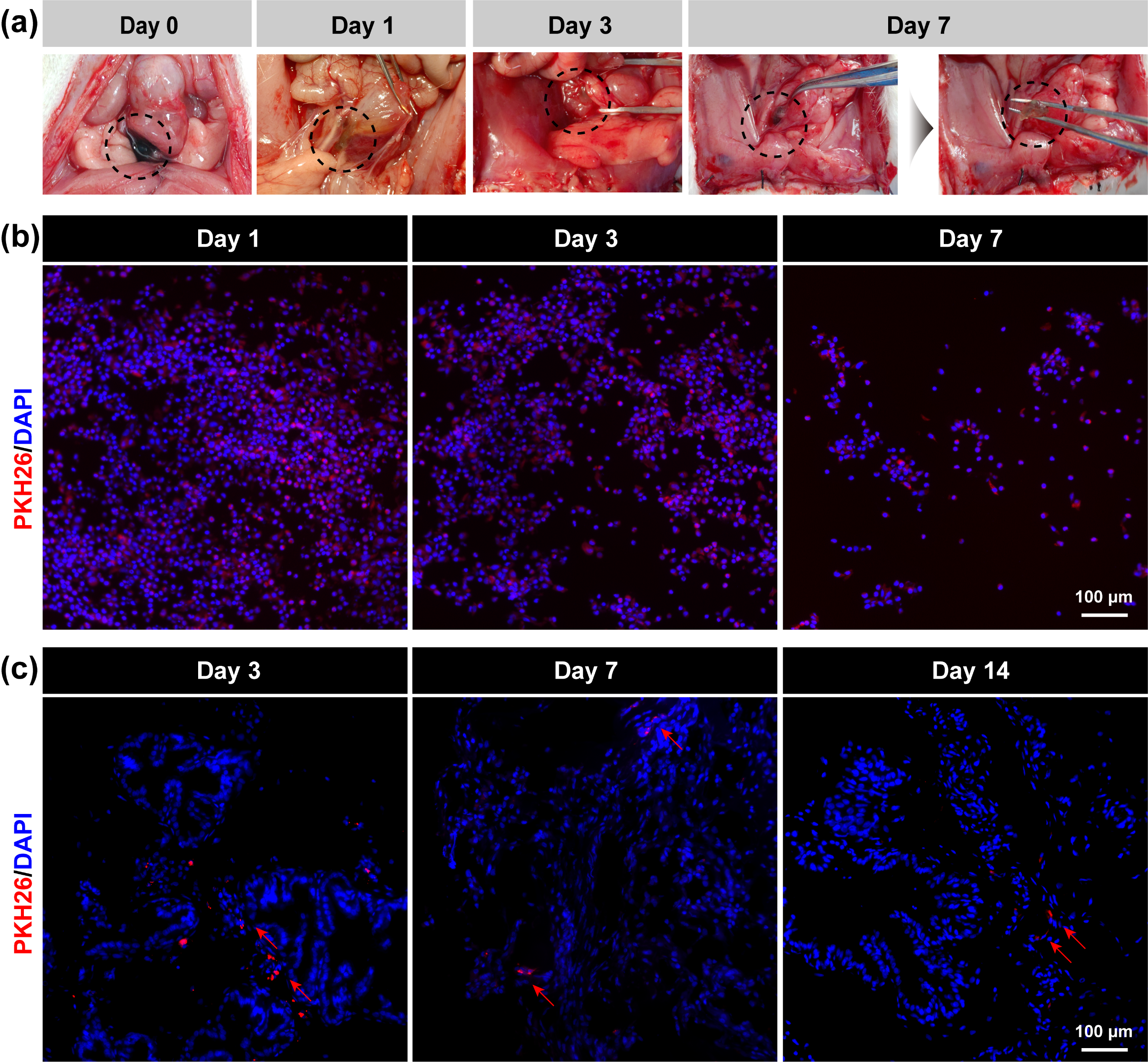


**Figure S7.** (a) Adhesive property of hydrogels *in vivo*. (b) PKH26 labeled ADSC in the residual hydrogels. (c) The monitor of PKH26 labeled ADSC in MPG tissues.


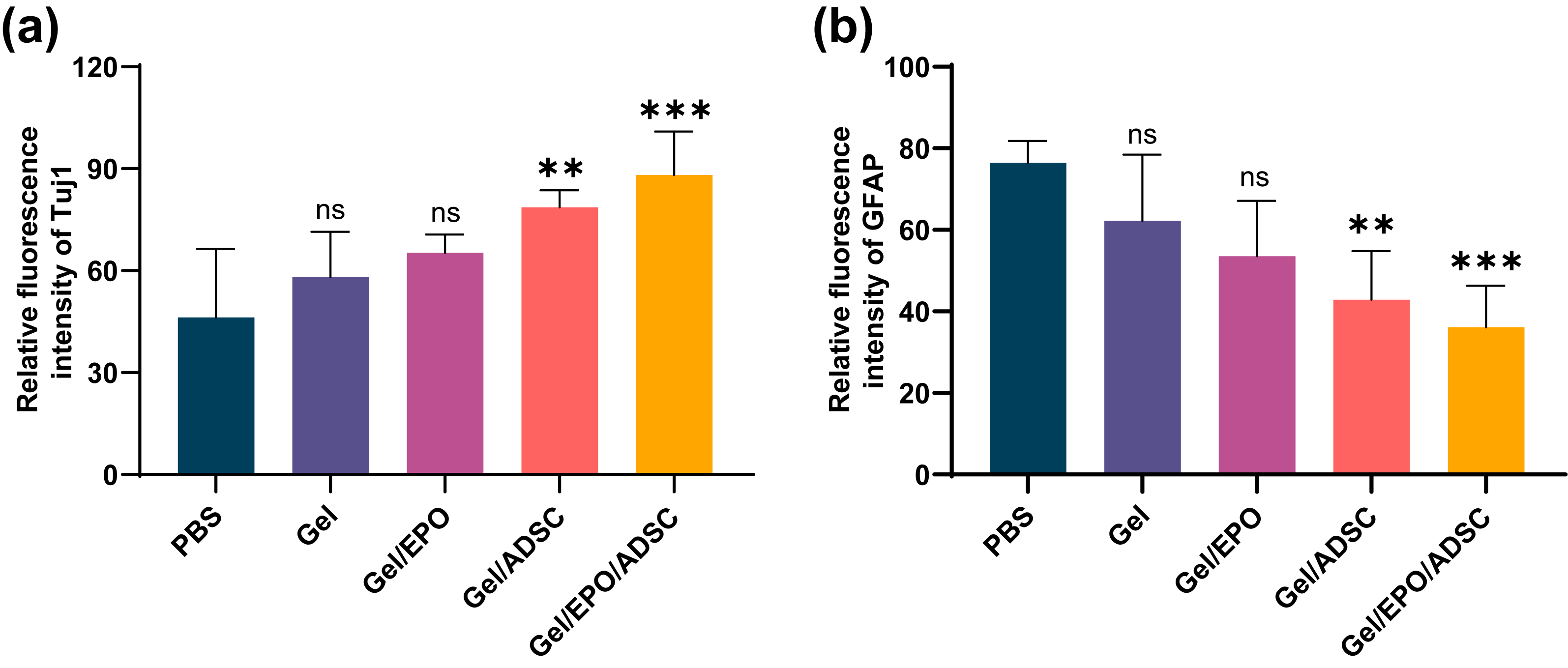


Figure S8. Relative fluorescence intensity of Tuj1 (a) and GFAP (b) among groups. One-way ANOVA followed by post hoc Bonferroni analysis. (**) P < 0.01, and (***) P < 0.001. Note that Gel represents GelMA/HADA/CMCDA hydrogel.
